# Deficiency of IL-20 receptor subunit A decreases enterovirus A71 lethality in mice by increasing M1 macrophage polarization and cytokine production

**DOI:** 10.3389/fimmu.2025.1700154

**Published:** 2026-01-02

**Authors:** Cheng-Huei Hung, Yi-Ling Hsiao, Yi-Ping Tsai, Ming-Shi Chang, Ching-Chuan Liu, Li-Chiu Wang, Shun-Hua Chen

**Affiliations:** 1 Institute of Basic Medical Sciences, College of Medicine, National Cheng Kung University, Tainan, Taiwan; 2 Department of Microbiology and Immunology, College of Medicine, National Cheng Kung University, Tainan, Taiwan; 3 Department of Biochemistry and Molecular Biology, College of Medicine, National Cheng Kung University, Tainan, Taiwan; 4 Department of Pediatrics, National Cheng Kung University Hospital, National Cheng Kung University, Tainan, Taiwan; 5 Department of Post-Baccalaureate Medicine, College of Medicine, National Chung Hsing University, Taichung, Taiwan; 6 Center of Infectious Disease and Signaling Research, National Cheng Kung University, Tainan, Taiwan

**Keywords:** IL-20RA cytokines, enterovirus A71, IL-10, IL-12, IFN-γ, macrophage polarization

## Abstract

**Introduction:**

Enterovirus A71 (EV-A71) can cause fatal disease accompanied by increased cytokines, including IL-10, IL-12, and IFN-γ, which are mutually regulated. IFN-γ is induced to protect mice from EV-A71 infection, but its regulation remains unclear. The IL-10 family cytokines, IL-19, IL-20, and IL-24, which signal through a two-subunit receptor complex containing IL-20 receptor subunit A (IL-20RA), are designated as IL-20RA cytokines. IL-20RA cytokines are known to regulate IFN-γ and IL-10 *in vitro*. We designed this study to investigate the interaction and role of IL-20RA cytokines in viral infection *in vivo*, which remain unknown.

**Methods:**

Plasma from healthy donors and EV-A71-infected patients was analyzed to detect IL-20RA cytokines. Wild-type (WT) and *IL-20RA* knockout (*IL-20RA*^-/-^) mice, as well as isolated T cells and macrophages, were used for functional studies.

**Results:**

In plasma samples, IL-19 was detectable in healthy controls, and EV-A71 infection increased IL-19 levels in infected patients. In sera of WT mice, IL-20RA cytokines, but not IL-10, IL-12, or IFN-γ, were detected in mock-infected animals, and EV-A71 infection significantly increased IL-19 and slightly increased IL-20 levels. Compared with WT mice, *IL-20RA*^-/-^ mice were resistant to EV-A71 infection, with reduced viral loads in peripheral organs, such as the spleen. In sera of infected mice, IL-20RA deficiency sequentially reduced IL-10 levels but increased IL-12 and IFN-γ levels. Abundant T cells expressed IL-10 in splenocytes of infected WT mice, whereas abundant macrophages expressed IL-12 and IFN-γ in splenocytes of infected *IL-20RA*^-/-^ mice. Notably, IL-20RA deficiency reduced M2 macrophages but increased M1 macrophages in splenocytes of infected mice. *In vitro*, treatment of leukocytes isolated from WT mice with IL-19 or IL-20, but not IL-24, increased IL-10 production in CD4 T cells and reduced IL-12 production in macrophages.

**Discussion:**

EV-A71 infection enhances IL-20RA cytokines, which then increase viral loads and aggravate disease severity in WT mice by elevating the T cell–IL-10–M2 macrophage axis and suppressing the protective M1 macrophage–IL-12–macrophage–IFN-γ axis. This represents a previously unreported mechanism.

## Introduction

Enterovirus A71 (EV-A71) infects humans through the fecal–oral route and can induce mild illness, such as fever, herpangina, and hand-foot-and-mouth disease (1–3). This virus can also cause severe symptoms, including brainstem encephalitis combined with pulmonary edema, which often leads to death or long-term neurological sequelae, especially in young children (1). EV-A71 is an RNA virus with a positive-sense, single-stranded RNA genome and a capsid (4). EV-A71 is a neurotropic virus capable of infecting neurons and has caused deadly outbreaks in the Asia–Pacific region, including Taiwan, for decades (2–4). Currently, specific and effective antivirals are unavailable. Intravenous immunoglobulin (IVIG) has been used to treat infected patients with severe symptoms in countries such as Taiwan (5), but its specificity and efficacy are limited. Vaccines have been used in limited regions and require additional locations and years to validate their effectiveness (6–8).

Studies of plasma specimens from infected patients show that EV-A71 infection increases IL-10, IL-12, and IFN-γ (9, 10) with IL-10 detected before IFN-γ (10). Using a murine infection model, we previously demonstrated that EV-A71 induces IFN-γ, which protects the host from infection by reducing viral replication (11). Although IFN-γ plays a protective role during infection, its regulation during EV-A71 infection remains unclear. IFN-γ can be upregulated by several mechanisms. It can enhance its own expression in an autocrine manner (12). It can promote the polarization of M1 macrophages, which express CD86 and MHC-II on the cell surface and produce cytokines, such as IL-12 (13, 14). IL-12 and IFN-γ can mutually amplify each other (15, 16). However, IFN-γ can also be downregulated by different mechanisms. For example, IL-10 inhibits IL-12 (17, 18). IL-10 can promote the polarization, and M2 macrophages primarily produce IL-10 (13, 14). Additionally, IL-19 or IL-20, but not IL-24, can increase IL-10 and decrease IFN-γ in T cells, as demonstrated by *in vitro* studies (19–21). The interaction between IFN-γ and IL-10, IL-12, IL-19, and IL-20 remains unclear.

IL-10, IL-19, and IL-20 belong to the IL-10 family, which contains nine members: IL-10; the IL-20 subfamily members IL-19, IL-20, IL-22, IL-24, and IL-26; and the distantly related cytokines IL-28A, IL-28B, and IL-29, which are also classified as type III interferons IFN-λ2, IFN-λ3, and IFN-λ1, respectively (22, 23). The IL-20 subfamily members utilize a heterodimeric receptor complex with two subunits to transduce signals. IL-19, IL-20, and IL-24 share the receptor composed of IL-20RA (also called IL-20R1 or IL-20Rα) and IL-20RB. IL-20 and IL-24 can also signal through IL-22RA1 paired with IL-20RB. IL-26 signals through IL-20RA and IL-10RB (22). Because IL-19, IL-20, IL-24, and IL-26 signal through IL-20RA, they are designated as “IL-20RA cytokines” in the present study. Humans express all four IL-20RA cytokines, whereas mice express only IL-19, IL-20, and IL-24 (22). IL-20RA cytokines are known to target T cells, macrophages, monocytes, and epithelial cells (21–24).

The interaction of IL-20RA cytokines with viral infection remains unknown, likely because these cytokines share receptors, functions, and activities. *IL-20RA* knockout (*IL-20RA^-/-^*) mice have been generated (25). These mice appear healthy and are therefore available for study. Among IL-20RA cytokines in *IL-20RA*^-/-^ mice, signaling by IL-19 is completely blocked, whereas IL-20 and IL-24 can still signal through IL-22RA1 paired with IL-20RB. We designed this study to investigate the interaction and role of IL-20RA cytokines in viral infection *in vivo* using murine models comparing wild-type (WT) and *IL-20RA*^-/-^ mice. We found that IL-20RA cytokines induce macrophage polarization and aggravate viral infections.

## Materials and methods

### Cell, virus, and mice

The human muscular (rhabdomyosarcoma, RD) cell line was maintained and propagated according to the instructions of the American Type Culture Collection. EV-A71 strain M2, a mouse-adapted virus, was propagated and titrated using RD cell monolayers as previously described (26). WT C57BL6/J mice and C57BL6/J-derived *IL-20RA*^-/-^ mice (25) were bred and maintained under specific pathogen-free conditions in the Laboratory Animal Center of our university.

### Infection of mice, tissue collection, and treatments of mice with IFN-γ antibody or liposomes

Twelve- to 14-day-old WT and *IL-20RA^-/-^* mice were infected with 1–2 × 10^6^ plaque-forming units (PFU)/mouse of EV-A71 by intraperitoneal inoculation and monitored for survival and disease scores for 12–14 days. The disease score was graded as follows: 0, healthy; 1, ruffled hair; 2, weakness in hind limbs; 3, paralysis in a single hind limb; 4, paralysis in both hind limbs; and 5, death.

Mice were anesthetized, and blood was collected. Mice were then perfused by intracardial injection of ice-cold phosphate-buffered saline (PBS) containing 0.01 M EDTA and 0.2% bovine serum albumin, and tissues were harvested. Blood samples were processed into serum, frozen at −80°C, and sonicated. Mouse organs and tissues were frozen, thawed, homogenized in 1 mL PBS, frozen, thawed, sonicated, and centrifuged at 13,000 rpm for 15 min at 4°C. The resulting sera and organ/tissue supernatants were assayed for viral titers by plaque assay on RD cell monolayers as previously described (26). *IL-20RA^-/-^* mice were treated with 25 μg of anti–IFN-γ antibody (clone R4-6A2, Bio X Cell) or normal rat IgG (Sigma), or with 50 μL of control liposomes (FormuMax) or clodronate liposomes (FormuMax), by intraperitoneal injection one day before infection and on days 1, 3, 5, and 7 post-infection.

### Treatments of mouse leukocytes with IL-19, IL-20, or IL-24 *in vitro*

Spleens were collected from uninfected, euthanized WT mice and processed into single-cell suspensions. CD4 T cells were isolated to 90% purity by positive selection using anti-CD4 magnetic beads (BioLegend) according to the manufacturer’s instructions. Mouse CD4 T cells were incubated in the absence or presence of IL-19, IL-20, or IL-24 (R&D Systems; 100 ng/mL) in RPMI-1640 medium containing 10% fetal bovine serum at 37°C and 5% CO_2_ and were harvested 8 and 24 h after cytokine treatment.

Peritoneal macrophages were harvested from uninfected, euthanized WT or *IL-20RA^-/-^* mice. More than 90% of the collected cells were positive for F4/80, a marker specific for mouse macrophages, as determined by immunofluorescence staining as previously described (12). Macrophages were incubated in the absence or presence of IL-19, IL-20, or IL-24 (100 ng/mL) for 3 days and were stimulated or not with poly I:C (InvivoGen; 100 ng/mL) for 24 h in Dulbecco’s Modified Eagle Medium containing 10% fetal bovine serum at 37°C and 5% CO_2_. Culture supernatants of CD4 T cells and macrophages were harvested to quantify IL-10 and IL-12, respectively, by enzyme-linked immunosorbent assay (ELISA).

The remaining cells were processed to extract total RNA using the GENEzol TriRNA Pure Kit (Geneaid) according to the manufacturer’s instructions. Total RNA from CD4 T cells and macrophages was subjected to RT-PCR to quantify mRNA encoding IL-10 or IL-12, respectively.

### Quantitative real-time RT-PCR

After reverse transcription with reverse primers, the synthesized cDNA was used for quantitative real-time PCR with forward and reverse primers. PCR was performed at 95°C for 10 min followed by 40 cycles of denaturation (95°C, 15 s) and annealing (60°C, 1 min) using the Fast SYBR Green Master Mix kit (Thermo Fisher Scientific). The threshold cycle (*C*_T_) of each product was determined, normalized to the internal control (β-actin), and shown as Δ*C*_T_. All results are presented as the ratio to β-actin calculated as 2^−Δ^*^C^*^T^. Primers for mRNA encoding IL-10 (forward 5’-ATA ACT GCA CCC ACT TCC CA-3’ and reverse 5’-GGG CAT CAC TTC TAC CAG GT-3’), β-actin (forward 5’-AAC CCT AAG GCC AAC CGT GAA AAG ATG ACC-3’ and reverse 5’-CCA GGG AGG AAG AGG ATG CGG C-3’), IL-12 p35 (forward 5’-AGG ACT TGA AGA TGT ACC AG-3’ and reverse 5’-CTA TCT GTG TGA GGA GGG-3’), and IL-12 p40 (forward 5’-GGA AGC ACG GCA GCA GAA TAA-3’ and reverse 5’-CTT GAG GGA GAA GTA GGA ATG-3’) were used.

### Collection of human specimens

Plasma specimens were collected from EV-A71-infected patients admitted to National Cheng Kung University Hospital during epidemics and from age-matched healthy control subjects. EV-A71 infection was defined as the isolation and typing of the virus from at least one site (throat swab, stool, cerebrospinal fluid, or other) with a negative bacterial culture.

Brainstem encephalitis was defined as an illness characterized by myoclonus, ataxia, nystagmus, oculomotor palsies, and bulbar palsy in various combinations, with or without neuroimaging. Pulmonary edema was defined as respiratory distress with tachycardia, tachypnea, rales, and frothy sputum, together with a chest radiograph showing bilateral pulmonary infiltrates without cardiomegaly.

### Cytokine measurement by ELISA

Brains harvested from mice were frozen, homogenized in 1 mL PBS containing a protease inhibitor cocktail (Sigma-Aldrich), and centrifuged. Mouse sera, brain supernatants, and T cell or macrophage culture supernatants were assayed using commercially available ELISA kits for IL-10 (BioLegend), IL-12 (BioLegend), IL-19 (eBioscience), IL-20 (R&D Systems), IL-24 (Elabscience), or IFN-γ (R&D Systems), with detection limits of 2.7, 0.5, 62.5, 6.4, 18, and 2 pg/mL, respectively, according to the manufacturers’ instructions.

Human plasma samples were assayed using commercially available ELISA kits for IL-10 (BioLegend), IL-12 (BioLegend), IL-19 (Abcam), IL-20 (Abcam), and IFN-γ (BioLegend), with detection limits of 2, 2, 0.6, 4.2, and 1.3 pg/mL, respectively, according to the manufacturers’ instructions.

### Flow cytometry

Leukocytes were isolated from mouse spleens as previously described (27) and blocked with anti-CD16/CD32 antibody (clone 93; BioLegend) against Fc receptors to prevent nonspecific binding. The leukocytes were stained with antibodies against CD3 (clone 17A2, BD Biosciences), CD11b (clone M1/70, BD Biosciences), CD11c (clone HL3, BD Biosciences), CD19 (clone 1D3, BD Biosciences), CD45 (clone 30-F11, BioLegend), CD86 (clone GL1, BD Biosciences), CD206 (clone C068C2, BioLegend), CD335 (clone NKp46, BD Biosciences), arginase-1 (clone W21047I, BioLegend), F4/80 (clone CI:A3-1, Bio-Rad), Ly6G (clone 1A8, BD Biosciences), or MHC-II (clone M5/114.15.2, BD Biosciences) on the cell surface.

Cells were then fixed and permeabilized with the Cytofix/Cytoperm kit (BD Biosciences) before staining for intracellular IFN-γ (clone XMG1.2, BioLegend), IL-10 (clone JES5, BioLegend), or IL-12 (clone C15.6, BioLegend). Samples were analyzed using a CytoFLEX flow cytometer (Beckman Coulter).

### Statistical analyses

Data are expressed as mean ± SEM. For statistical comparison, levels of mouse cytokines, tissue viral titers, flow cytometry results, and human cytokines were analyzed by the Mann–Whitney *U* test; mouse disease scores were analyzed by two-way ANOVA; and mouse survival rates were analyzed by the log-rank test. *In vitro* data were analyzed by the Student’s *t* test. All *P* values represent two-tailed significance tests. A *P* value of <0.05 was considered statistically significant.

## Results

### Among mouse IL-20RA cytokines, EV-A71 infection significantly increases mouse serum and brain IL-19 levels and slightly increases mouse serum IL-20 and IL-24 levels

**Figure 1:**
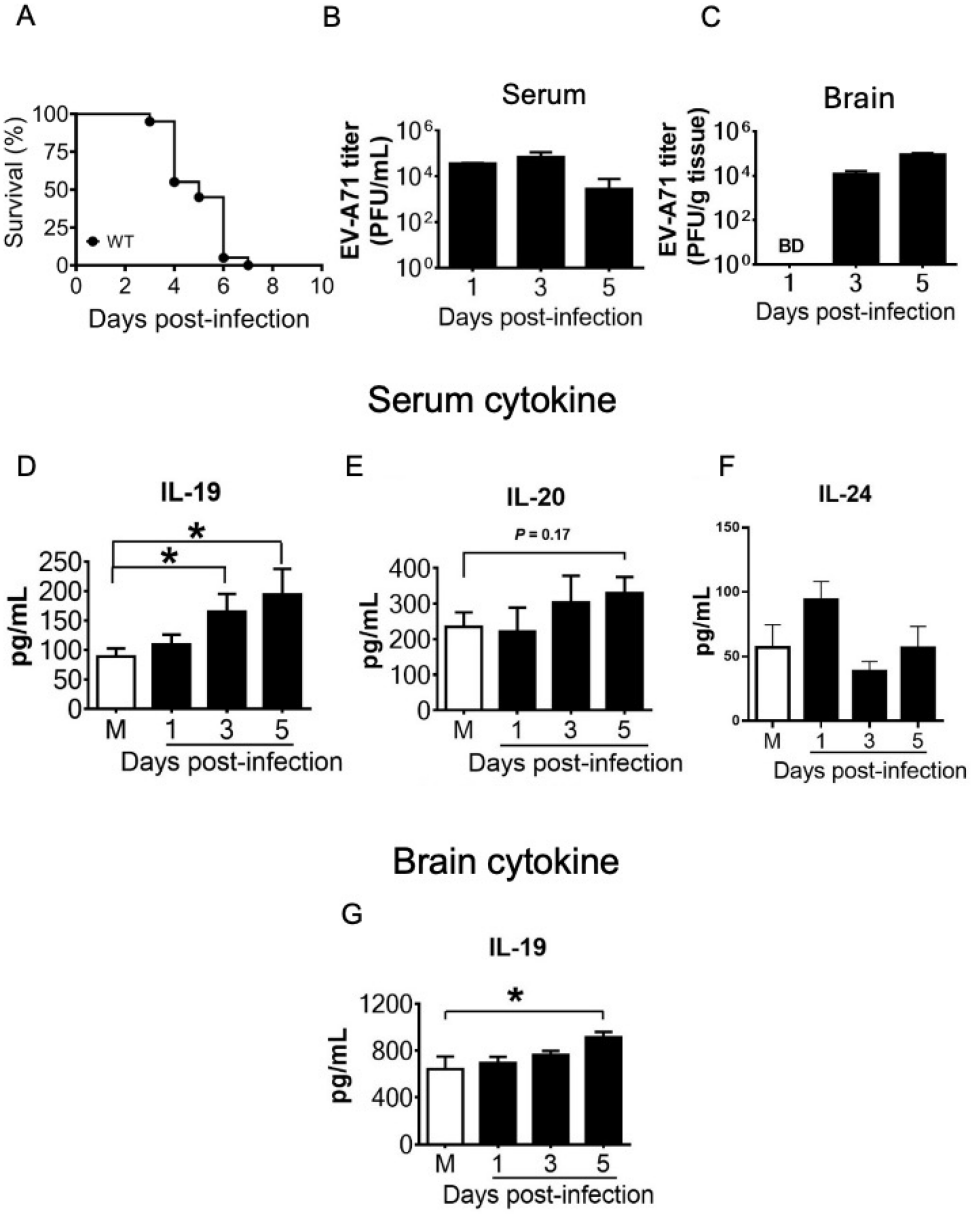
Levels of viral titers and IL-20RA cytokines in mice. **(A)** Wild-type mice (WT, *n* = 10) were infected with EV-A71 (1 × 10^6^ PFU/mouse) and monitored for survival. The sera **(B, D-F)** and brains **(C, G)** of mice infected with EV-A71 and collected on 1, 3, or 5 days post-infection (black bars) or of mice mock-infected and collected on the day of infection (M, white bars) were assessed to determine the levels of virus by the plaque assay **(B, C)** and the indicated cytokines by ELISA **(D-G)**. Data show means + SEM of ≥5 samples per group. *P < 0.05. B.D. stands for below the detection limit.

Few reports have investigated the influence of viral infection on IL-20RA cytokine expression *in vivo*, so we addressed this issue using a murine model. WT mice were infected with EV-A71 (1 × 10^6^ PFU/mouse) by intraperitoneal inoculation to induce systemic infection, and all infected mice succumbed to death 7 days post-infection (d.p.i.) (**Figure 1A**). Mouse sera and brains were harvested for analyses of infectious virus by plaque assay on 1, 3, and 5 d.p.i., during the window in which mice were still available and based on our previous findings showing detectable EV-A71 can be detected (27). Virus was detected in the serum from 1 to 5 d.p.i. and in the brain on 3 and 5 d.p.i. (**Figures 1B, C**).

Mouse samples were also subjected to ELISA to assess IL-20RA cytokines. In sera, all three mouse IL-20RA cytokines, IL-19, IL-20, and IL-24, were detected in mock-infected mice, indicating constitutive expression (**Figures 1D–F**). EV-A71 infection enhanced IL-19 levels from 1 to 5 d.p.i., with significant increases on 3 and 5 d.p.i., and slightly increased IL-20 levels on 3 and 5 d.p.i. and IL-24 levels on 1 d.p.i. (**Figures 1D–F**). In brains, IL-19 was detected in mock-infected mice, and EV-A71 infection increased IL-19 levels on 5 d.p.i. (**Figure 1G**). However, brain IL-20 and IL-24 levels were below detection in both mock-infected and infected mice from 1 to 5 d.p.i.

### IL-20RA deficiency decreases viral loads in peripheral organs and EV-A71 lethality

**Figure 2:**
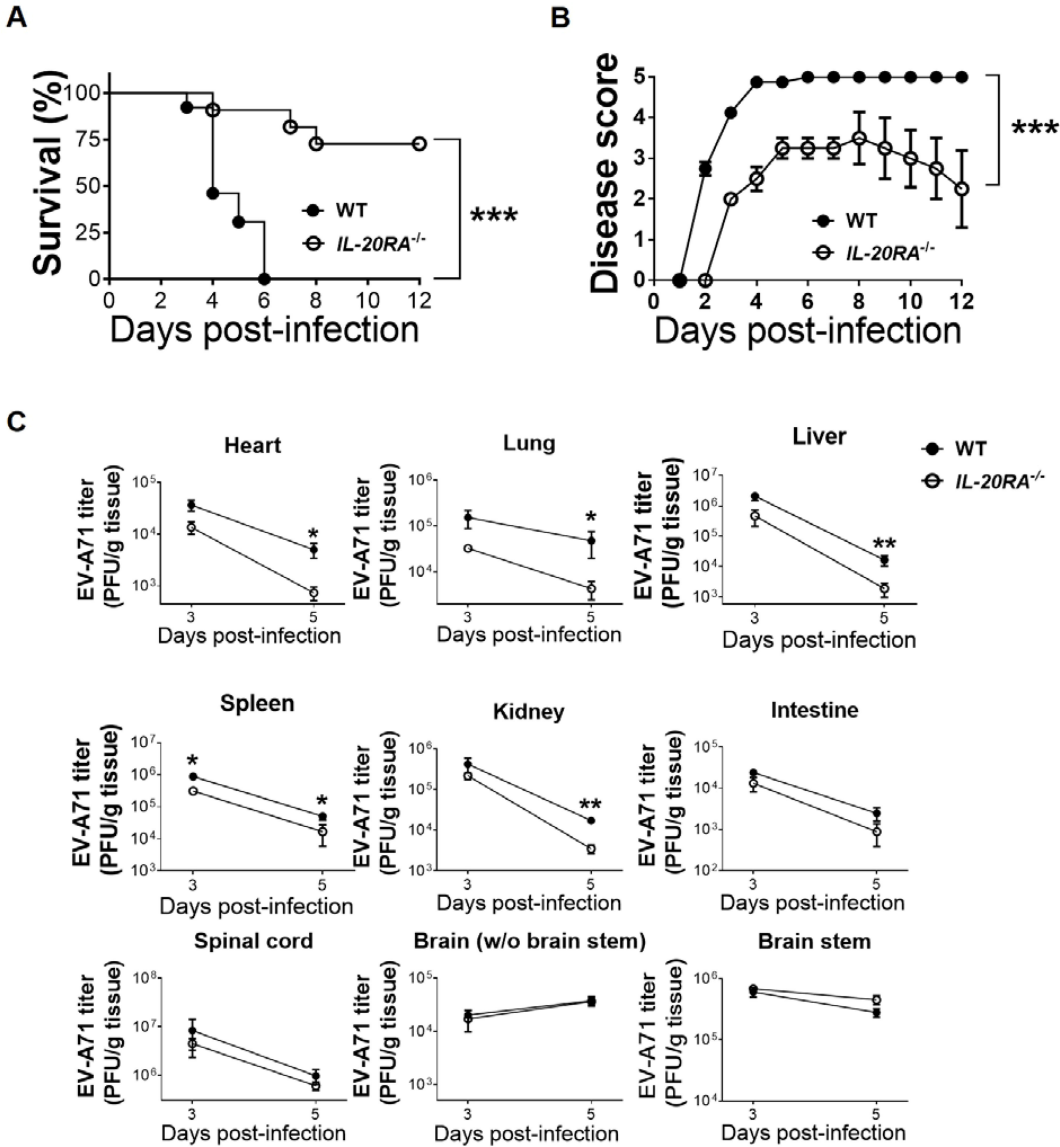
IL-20RA deficiency reduces viral loads in peripheral organs and EV-A71 lethality of mice. The survival rates **(A)**, disease scores **(B)**, and organ/tissue viral loads **(C)** of infected WT mice (black circles) and *IL-20RA*^-/-^ mice (white circles) are shown. In panels **(A–C)***n* ≥ 10, *n* ≥ 10, and *n* = 6 per group or per group or data point, respectively. Data show means ± SEM in panels **(B, C)** *P < 0.05, **P < 0.01, ***P < 0.001, compared between WT and *IL-20RA*^-/-^ groups on the same day **(C)** or the indicated groups **(A, B)**.

Because all three mouse IL-20RA cytokines were detected in WT mice, we next investigated their significance in EV-A71 infection by comparing WT and *IL-20RA*^-/-^ mice. After infection, WT mice exhibited higher death rates and higher disease scores than *IL-20RA*^-/-^ mice (**Figures 2A, B**). Viral loads in peripheral organs, including the heart, lung, liver, spleen, and kidney, were higher in WT mice than in *IL-20RA*^-/-^ mice on 3 and/or 5 d.p.i. (**Figure 2C**). Viral loads in the intestine and central nervous system (CNS), including the spinal cord, brainstem, and brain excluding the brainstem, did not differ significantly between WT and *IL-20RA*^-/-^ mice on 3 and 5 d.p.i. (**Figure 2C**).

In subsequent experiments, we focused on peripheral organs rather than the CNS for two reasons. First, all IL-20RA cytokines were detected in the serum (periphery) but not in the CNS of both mock-infected and infected WT mice. Second, the major effect of IL-20RA deficiency on viral loads was observed in peripheral organs, not in the CNS.

We observed that EV-A71 infection significantly increased serum and brain IL-19 levels in WT mice. Mice with deletion of the gene encoding IL-19 have been generated and are viable and healthy (28). We therefore compared IL-19-deficient mice (kindly provided by Dr. Yasu-Taka Azuma, Osaka Prefecture University) with WT mice infected with EV-A71 (1 × 10^6^ PFU/mouse) by intraperitoneal inoculation. All infected IL-19-deficient mice (*n* = 10) and WT mice (*n* = 14) succumbed to death, suggesting that the effect of IL-20RA cytokines on EV-A71 infection may not be mediated by IL-19 alone. Because IL-19, IL-20, and IL-24 share biological activities (22), IL-20 and/or IL-24 may compensate for the loss of IL-19 in IL-19-deficient mice. These findings may explain the lack of survival differences between infected WT and IL-19-deficient mice and support the necessity of comparing WT and *IL-20RA*^-/-^ mice in subsequent studies.

### IL-20RA deficiency enhances the serum IFN-γ level of infected mice, and IFN-γ depletion increases peripheral organ viral loads and EV-A71 lethality in *IL-20RA*^-/-^ mice

IL-20RA cytokines can regulate cytokine expression (21–23, 29). As type I IFNs (IFN-α and IFN-β), IL-1β, and IL-6 have been shown to protect mice from EV-A71 infection (30–32), we monitored these cytokines and TNF-α using ELISA. The serum and/or brain levels of these cytokines detected in infected WT mice were not significantly lower than those of infected *IL-20RA*^-/-^ mice from 1 to 5 d.p.i. (**Supplementary Figure S1**).

**Figure 3:**
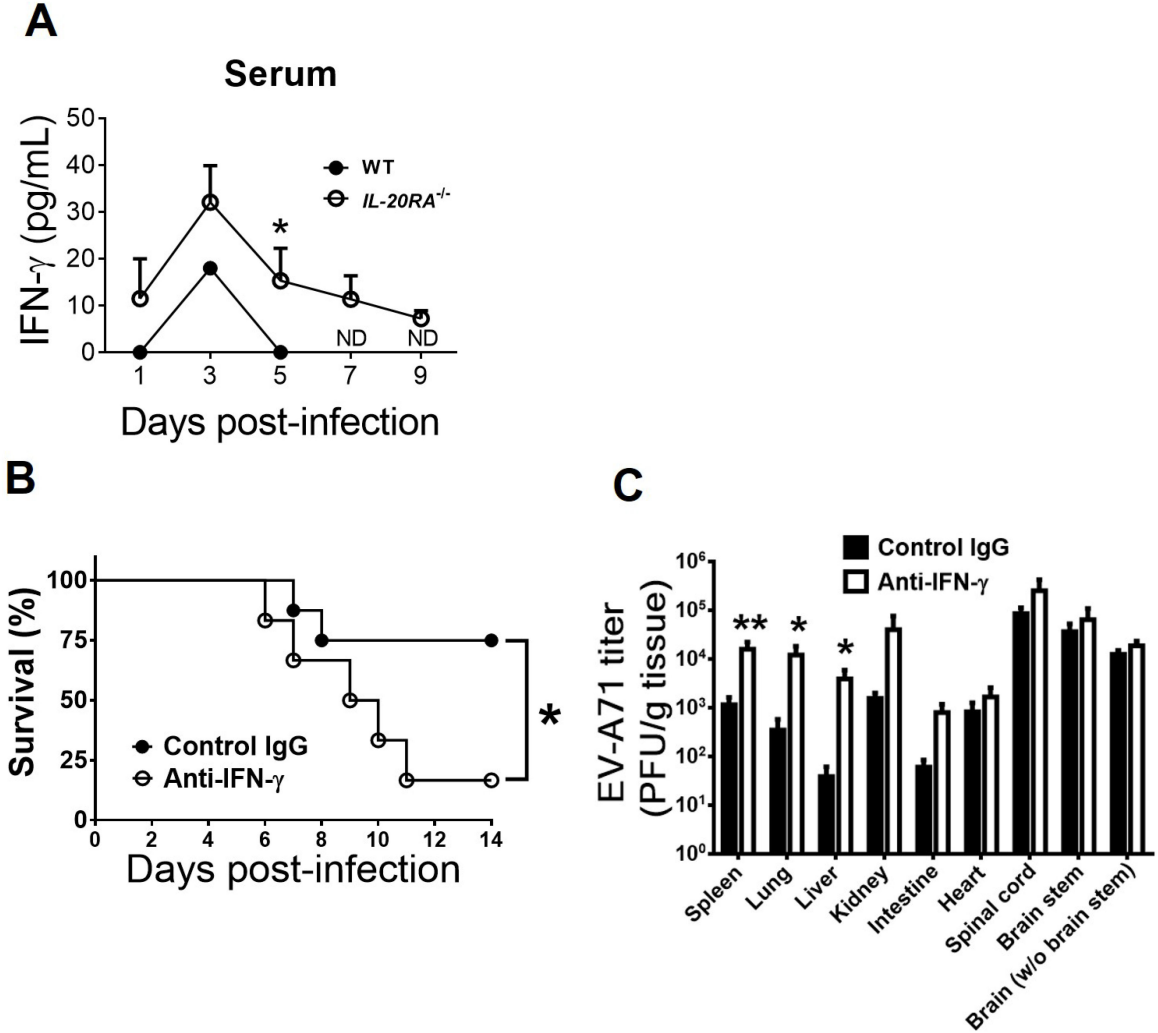
IL-20RA deficiency increases IFN-γ to reduce viral loads in peripheral organs and EV-A71 lethality of mice. **(A)** The sera of infected WT mice and *IL-20RA*^-/-^ mice were harvested to measure IFN-γ by ELISA. ND stands for “not done”, because samples were unavailable due to mouse death. The survival rates on indicated days **(B)** and organ/tissue viral loads on 7 days post-infection **(C)** of infected *IL-20RA*^-/-^ mice treated with the control or anti-IFN-γ antibody are shown. **(A-C)***n* ≥ 4, *n* = 8, and *n* = 8 per data point or group, respectively. Data show means + SEM **(A, C)** *P < 0.05 and **P < 0.01, compared between WT and *IL-20RA*^-/-^ groups on the same day **(A, C)** or the indicated groups **(B)**.

Previous *in vitro* studies showed that treatment with IL-20RA cytokines, IL-19 or IL-20, but not IL-24—decreases IFN-γ levels in primary human T cells *in vitro* (19, 20). More importantly, we previously demonstrated that EV-A71 induces IFN-γ to protect mice from infection by reducing viral replication (11). We therefore monitored IFN-γ in mice using ELISA. In mock-infected WT and *IL-20RA*^-/-^ mice, both serum and brain IFN-γ levels were below the limit of detection. EV-A71 infection induced IFN-γ in both genotypes, with reduced serum IFN-γ levels detected in WT mice compared with *IL-20RA*^-/-^ mice from 1 to 5 d.p.i., reaching statistical significance on 5 d.p.i. (**Figure 3A**). Brain IFN-γ levels in infected WT mice were slightly lower than those in infected *IL-20RA*^-/-^ mice on 3 and 5 d.p.i. (**Supplementary Figure S2**). All infected WT mice died on 6 d.p.i. (**Figure 2A**), so serum and brain samples could not be collected on 7 and 9 d.p.i.

To determine the significance of endogenous IFN-γ in *IL-20RA*^-/-^ mice, we depleted IFN-γ using a monoclonal antibody. IFN-γ depletion reduced the survival rate of infected *IL-20RA*^-/-^ mice and increased viral loads in peripheral organs (spleen, lung, and liver) on 7 d.p.i. (**Figures 3B, C**). These results indicate that endogenous IFN-γ protects *IL-20RA^-/-^* mice from EV-A71 infection. We next investigated the regulation of IFN-γ by IL-20RA cytokines during EV-A71 infection *in vivo*, as few studies address this issue.

### IL-20RA deficiency increases macrophages to express IFN-γ in infected mice, and macrophage depletion increases the death and serum IFN-γ level of infected *IL-20RA*^-/-^ mice

**Figure 4:**
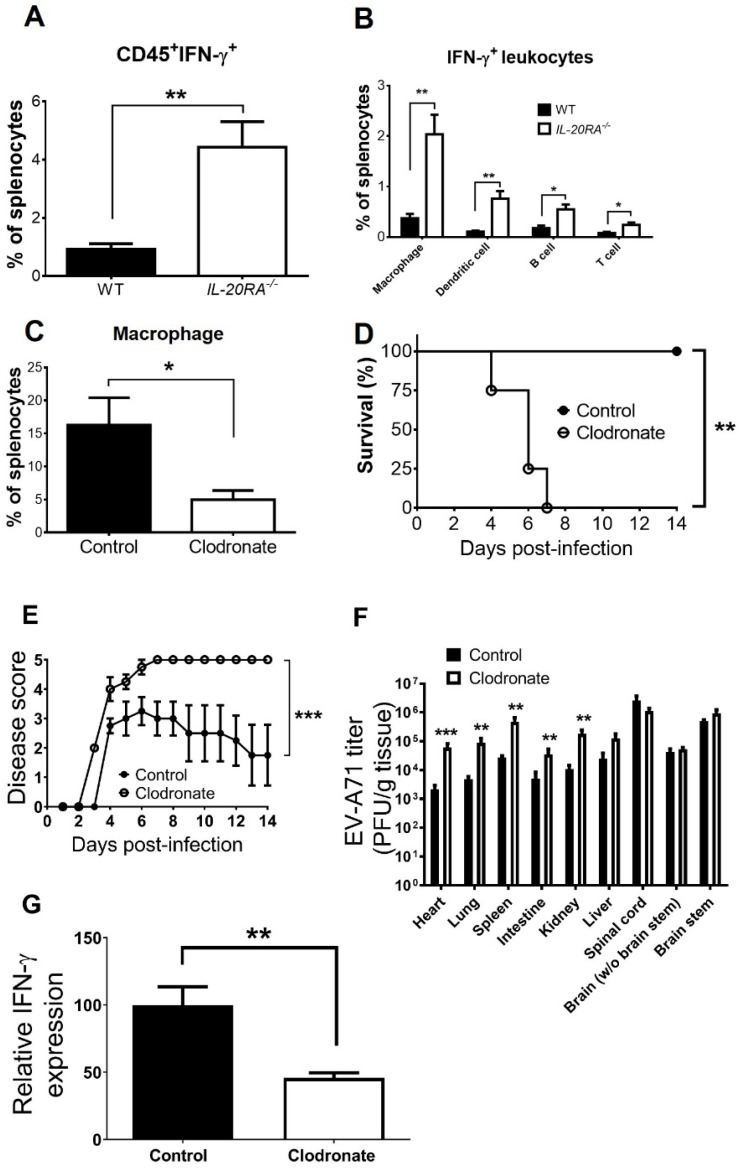
IL-20RA deficiency increases IFN-γ- expressing macrophages to reduce viral loads in peripheral organs and EV-A71 lethality of mice. The spleens of infected WT and *IL-20RA*^-/-^ mice were harvested on 5 days post-infection and processed to quantify splenocytes expressing leukocyte markers **(A)** CD45 plus **(B)** macrophages, dendritic cells, B cells, or T cells on the cell surface and IFN-γ in cells. The spleens of mock-infected *IL-20RA*^-/-^ mice treated with control or clodronate liposomes for one day were monitored for macrophage levels **(C)**. The survival rates **(D)** and disease scores **(E)** on indicated days and organ/tissue viral loads **(F)** as well as serum IFN-γ protein levels **(G)** on 5 days post-infection of infected *IL-20RA*^-/-^ mice treated with control or clodronate liposomes are shown. Sample sizes per group, *n* = 5 **(C)**, *n* = 7 **(D, E)**, and *n* = 6 for the rest of panels. Except panel **(D)**, data show means + or ± SEM. *P < 0.05; **P < 0.01; ***P < 0.001, compared between control and clodronate groups **(F)** or the indicated groups in the rest of panels.

IFN-γ is mainly expressed by leukocytes, and the spleen is composed of leukocytes. Moreover, abundant EV-A71 was detected in the spleen of infected mice, and both IL-20RA deficiency and IFN-γ depletion affected viral loads in the spleen (**Figures 2C**, **3C**). As IL-20RA deficiency increases the serum IFN-γ level of infected mice on 5 d.p.i., we therefore monitored splenocytes collected on 5 d.p.i. to quantify leukocytes expressing IFN-γ via staining CD45, a pan-leukocyte marker, on the cell surface and IFN-γ inside the cells by flow cytometry (gating strategy shown in **Supplementary Figure S3**). In mock-infected WT and *IL-20RA*^-/-^ mice, CD45^+^IFN-γ^+^ cells (leukocytes expressing IFN-γ) were minimal (data not shown). EV-A71 infection increased the percentages of CD45^+^IFN-γ^+^ cells in both WT and *IL-20RA*^-/-^ mice, with a reduced percentage of the cells detected in WT mice when compared to *IL-20RA*^-/-^ mice (**Figure 4A**). Macrophages, dendritic cells, B cells, and especially T cells can produce IFN-γ (33). We further quantified these leukocytes expressing IFN-γ in splenocytes of infected mice on 5 d.p.i. Notably, we found that in infected *IL-20RA*^-/-^ mice, a high percentage of (CD45^+^IFN-γ^+^CD11b^+^F4/80^+^) macrophages, followed by (CD45^+^IFN-γ^+^CD11c^+^) dendritic cells, (CD45^+^IFN-γ^+^CD19^+^) B cells, and (CD45^+^IFN-γ^+^CD3^+^) T cells, expressed IFN-γ (**Figure 4B**). Moreover, the percentages of all these four types of leukocytes expressing IFN-γ in the spleen of infected *IL-20RA*^-/-^ mice were higher than those of infected WT mice (**Figure 4B**).

As a high percentage of macrophages expressed IFN-γ in infected *IL-20RA*^-/-^ mice (**Figure 4B**), we assessed the importance of macrophages for *IL-20RA*^-/-^ mice to fight EV-A71 infection by depleting macrophages in *IL-20RA^-/-^* mice with the treatment of liposomes containing clodronate (34). Clodronate liposome treatment efficiently depleted macrophages by ~70%, when compared to control liposomes, in the spleen of mock-infected *IL-20RA^-/-^* mice one day after treatment (**Figure 4C**). In infected *IL-20RA^-/-^* mice, clodronate liposome treatment increased the viral loads in peripheral organs (heart, lung, spleen, intestine, and kidney), with significant differences found on 5 d.p.i., as well as increased disease scores and death rate, but decreased the serum IFN-γ level by >50% on 5 d.p.i. when compared to control liposomes (**Figures 4D–G**). These results showed that macrophages produce IFN-γ in infected *IL-20RA^-/-^* mice and that macrophages protect *IL-20RA^-/-^* mice from EV-A71 infection.

### IL-20RA deficiency increases the levels of spleen macrophages expressing IL-12 and serum IL-12 in infected mice

**Figure 5:**
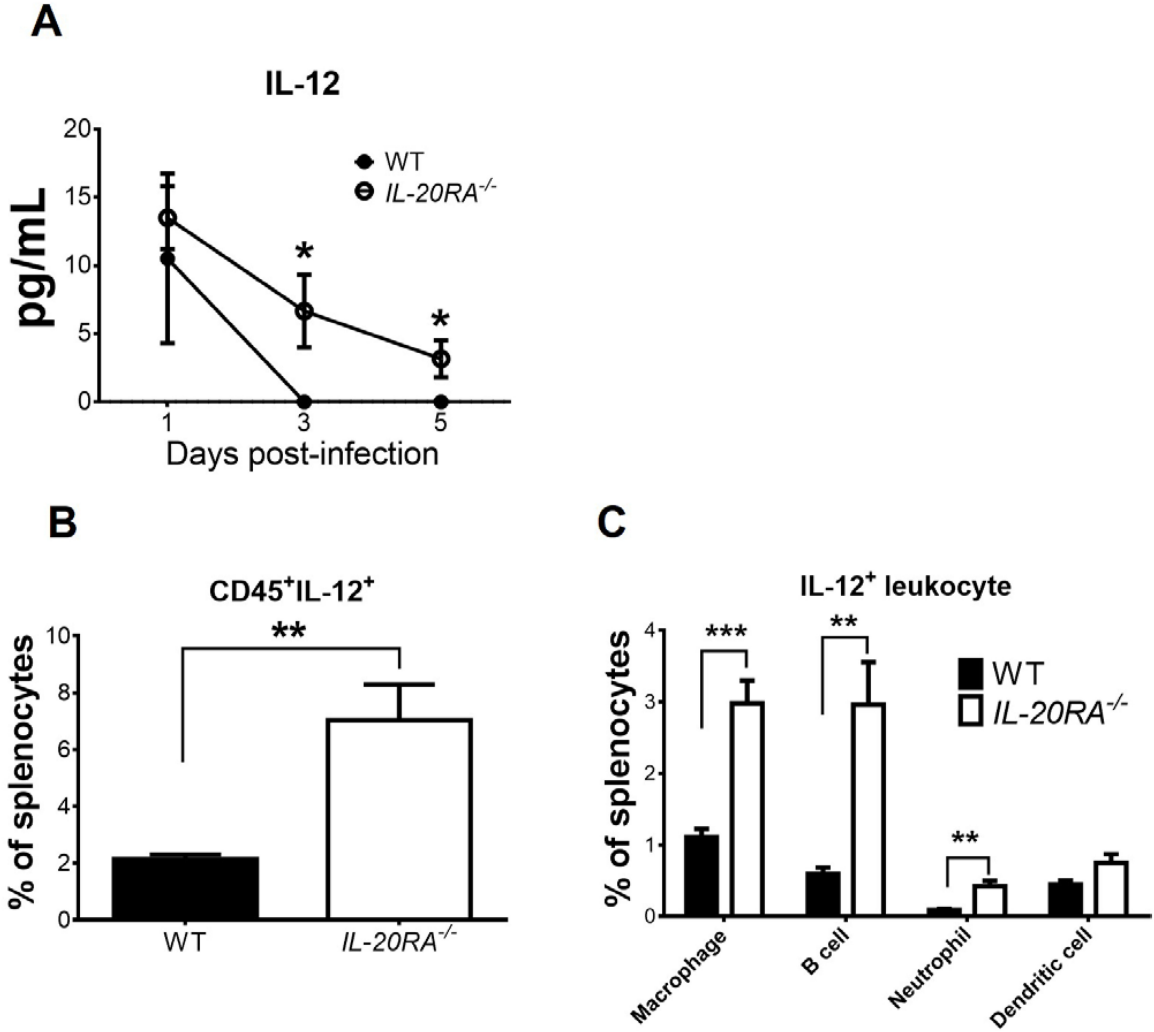
IL-20RA deficiency increases the levels of serum IL-12 and IL-12-expressing leukocytes in infected mice. **(A)** The sera of infected WT mice and *IL-20RA*^-/-^ mice were harvested to measure IL-12 by ELISA. The spleens of infected WT and *IL-20RA*^-/-^ mice were harvested on 3 days post-infection and processed to quantify splenocytes expressing leukocyte markers of **(B)** CD45 plus **(C)** macrophages, B cells, neutrophils, or dendritic cells on the cell surface and IL-12 in cells. Data show means ± or + SEM of 6 samples per data point or group. *P < 0.05; **P < 0.01; ***P < 0.001, compared between WT and *IL-20RA*^-/-^ groups on the same day **(A)** or the indicated groups **(B, C)**.

IL-12 can enhance macrophages to express IFN-γ (16), which can further amplify the production of both IL-12 and IFN-γ (15), so we monitored IL-12. In mock-infected WT and *IL-20RA*^-/-^ mice, the serum IL-12 levels were below detection. EV-A71 infection induced IL-12 in both WT and *IL-20RA*^-/-^ mice, with reduced serum IL-12 levels detected in WT mice when compared to *IL-20RA*^-/-^ mice from 1 to 5 d.p.i., with significant differences found on 3 and 5 d.p.i. (**Figure 5A**). The biggest difference in serum IL-12 levels of infected WT and *IL-20RA*^-/-^ mice was detected on 3 d.p.i. We therefore performed flow cytometry to quantify the leukocytes expressing IL-12 in splenocytes on 3 d.p.i. by staining leukocyte markers on the cell surface and IL-12 inside the cells. In mock-infected WT and *IL-20RA*^-/-^ mice, CD45^+^IL-12^+^ cells (leukocytes expressing IL-12) in splenocytes were minimal (data not shown). EV-A71 infection increased the percentages of CD45^+^IL-12^+^ cells in both WT and *IL-20RA*^-/-^ mice, with a reduced percentage detected in WT mice when compared to *IL-20RA*^-/-^ mice (**Figure 5B**). Dendritic cells, neutrophils, and especially macrophages as well as B cells can produce IL-12 (17). We further quantified these leukocytes expressing IL-12 in splenocytes of infected mice on 3 d.p.i. and found that in infected *IL-20RA*^-/-^ mice, high percentages of (CD45^+^IL-12^+^CD11b^+^F4/80^+^) macrophages and (CD45^+^IL-12^+^CD19^+^) B cells followed by (CD45^+^IL-12^+^CD11c^+^) dendritic cells and (CD45^+^IL-12^+^Ly6G^+^) neutrophils, expressed IL-12 (**Figure 5C**). Moreover, the percentages of macrophages, B cells, and neutrophils expressing IL-12 in infected *IL-20RA*^-/-^ mice were significantly higher than those of infected WT mice (**Figure 5C**).

### IL-20RA deficiency increases the level of M1 macrophages but decreases the level of M2 macrophages in the spleen of infected mice

**Figure 6:**
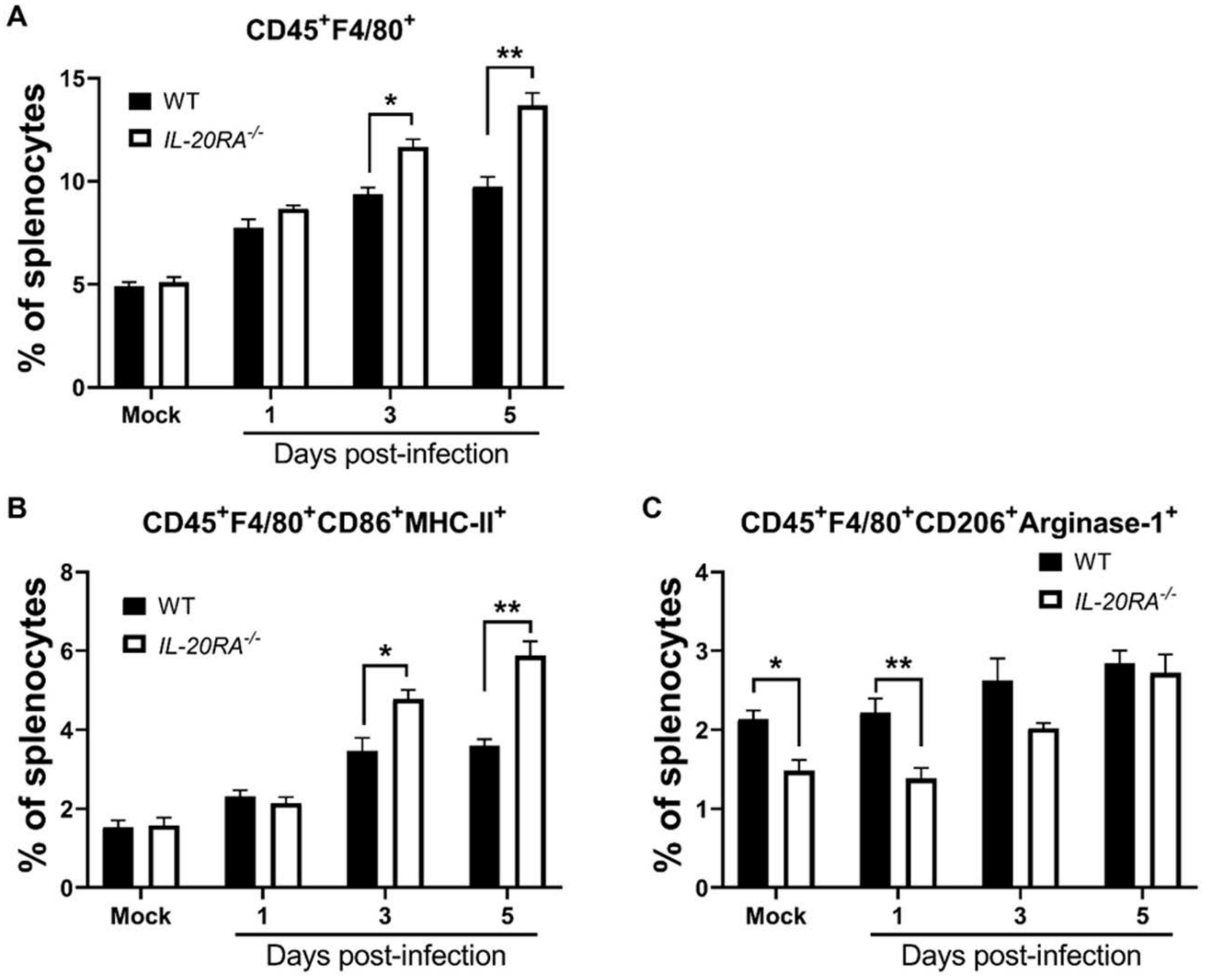
IL-20RA deficiency increases M1 macrophages in the spleen of infected mice. The spleens of WT and *IL-20RA*^-/-^ mice infected with EV-A71 for the indicated days or mock-infected (Mock) were harvested and processed to quantify splenocytes expressing markers of macrophages, CD45 and F4/80 **(A)**, M1 macrophages, CD45, F4/80, CD86, and MHC-II **(B)**, or M2 macrophages, CD45, F4/80, CD206, and Arginase-1 **(C)**. Data show means + SEM of 5 samples per group. *P < 0.05 and **P < 0.01.

IL-20RA deficiency increases the levels of serum IFN-γ and IL-12 as well as spleen macrophages expressing IFN-γ and IL-12 in infected mice (**Figures 3A**, **4B**, **5A, C**). As IFN-γ can promote the polarization of M1 macrophages, which produce cytokines such as IL-12 (13, 14), we quantified macrophages in mice. The percentages of (CD45^+^F4/80^+^) macrophages in the spleen of mock-infected WT and *IL-20RA*^-/-^ mice were comparable (**Figure 6A**). The percentages of (CD45^+^F4/80^+^) macrophages in the spleen of infected *IL-20RA*^-/-^ mice were significantly higher than those of infected WT mice on 3 and 5 d.p.i. (**Figure 6A**). The percentages of (CD45^+^F4/80^+^CD86^+^MHC-II^+^) M1 macrophages in the spleen of infected *IL-20RA*^-/-^ mice were higher than those of infected WT mice on 3 and 5 d.p.i. (**Figure 6B**). The percentages of (CD45^+^F4/80^+^CD206^+^Arginase-1^+^) M2 macrophages in the spleen of *IL-20RA*^-/-^ mice mock-infected or infected with virus for one day were lower than those of WT mice (**Figure 6C**). Overall, IL-20RA deficiency significantly promotes macrophage polarization toward the M1 phenotype in mice during infection.

### IL-20RA deficiency decreases the levels of serum IL-10 and spleen T cells expressing IL-10 in infected mice

**Figure 7:**
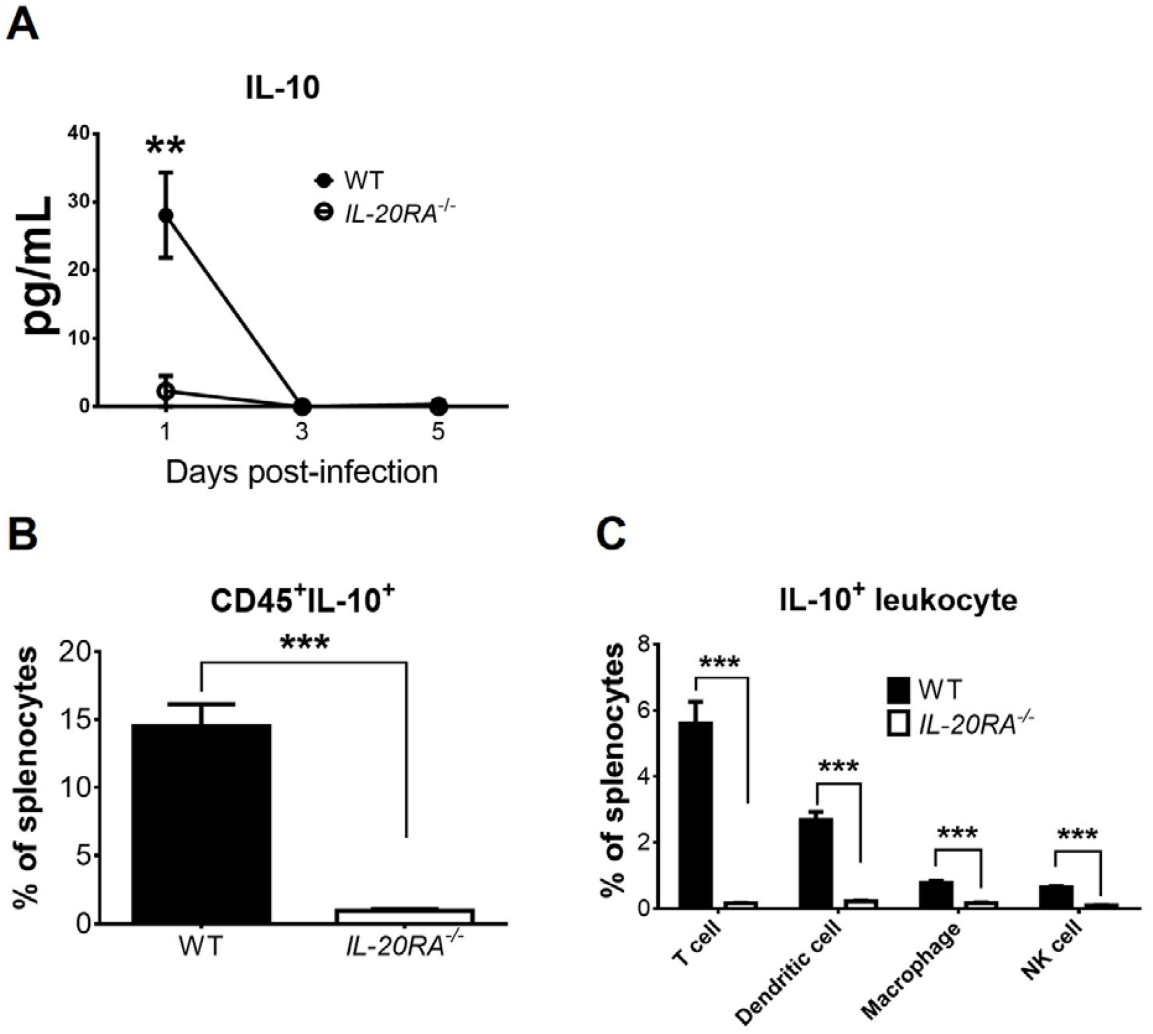
IL-20RA deficiency decreases levels of serum IL-10 and IL-10-expressing leukocytes in spleens of infected mice. **(A)** The sera of infected WT mice and *IL-20RA*^-/-^ mice were harvested to measure IL-10 by ELISA. The spleens of infected WT and *IL-20RA*^-/-^ mice were harvested on 1 day post-infection and processed to quantify splenocytes expressing leukocyte markers of **(B)** CD45 plus **(C)** T cells, dendritic cells, macrophages, or NK cells on the cell surface and IL-10 in cells. Data show means ± or + SEM of 6 samples per data point or group. **P < 0.01 and ***P < 0.001, compared between WT and *IL-20RA*^-/-^ mice on the same day **(A)** or the indicated groups **(B, C)**.

As IL-10 can promote the polarization of macrophages toward the M2 phenotype (13, 14) and inhibit IL-12 production in macrophages (18, 35–37), we therefore monitored IL-10. In mock-infected WT and *IL-20RA*^-/-^ mice, the serum IL-10 levels were below detection. EV-A71 infection induced IL-10 in both WT and *IL-20RA*^-/-^ mice, with an elevated serum IL-10 level detected in WT mice when compared to *IL-20RA*^-/-^ mice on 1 d.p.i. (**Figure 7A**). We performed flow cytometry to quantify the leukocytes expressing IL-10 in splenocytes on 1 d.p.i. by staining leukocyte markers on the cell surface and IL-10 inside the cells. In mock-infected WT and *IL-20RA*^-/-^ mice, the percentages of CD45^+^IL-10^+^ cells (leukocytes expressing IL-10) in splenocytes were minimal (data not shown). After infection, the percentage of CD45^+^IL-10^+^ cells in the splenocytes of WT mice was higher than that of *IL-20RA*^-/-^ mice (**Figure 7B**). Dendritic cells, macrophages, NK cells, and especially T cells can produce IL-10 (38). We further quantified these leukocytes expressing IL-10 in splenocytes of infected mice on 1 d.p.i. and found that in infected WT mice, a high percentage of (CD45^+^IL-10^+^CD3^+^) T cells followed by (CD45^+^IL-10^+^CD11c^+^) dendritic cells, (CD45^+^IL-10^+^CD11b^+^F4/80^+^) macrophages, and (CD45^+^IL-10^+^CD335^+^) NK cells expressed IL-10 (**Figure 7C**). Moreover, the percentages of all these four types of IL-10-expressing leukocytes in infected WT mice were higher than those of infected *IL-20RA*^-/-^ mice (**Figure 7C**).

CD4 T cells, especially Th2 cells, are major producers of IL-10 (39), and our additional results showed that the percentages of (CD45^+^IL-10^+^CD4^+^) cells, CD4 T cells expressing IL-10, in the splenocytes of infected WT mice were indeed higher than those of infected *IL-20RA*^-/-^ mice (**Supplementary Figure S4A**). We further identified the CD4 T cell subsets that produce IL-10. Our additional results showed that a high percentage of (CD45^+^IL-10^+^CD4^+^IL-4^+^GATA3^+^) Th2 cells, followed by (CD45^+^IL-10^+^CD4^+^CD25^+^Foxp3^+^) Treg cells and (CD45^+^IL-10^+^CD4^+^CXCR3^+^IFN-γ^+^) Th1 cells, expressed IL-10 in the splenocytes of infected WT mice (**Supplementary Figures S4B–D**). Moreover, the percentages of all these three subsets of CD4 T cells expressing IL-10 in infected WT mice were higher than those of infected *IL-20RA*^-/-^ mice (**Supplementary Figures S4B–D**).

### Treatment with IL-19 or IL-20 increases IL-10 production in mouse CD4 T cells, but reduces IL-12 production in mouse macrophages, *in vitro*

**Figure 8:**
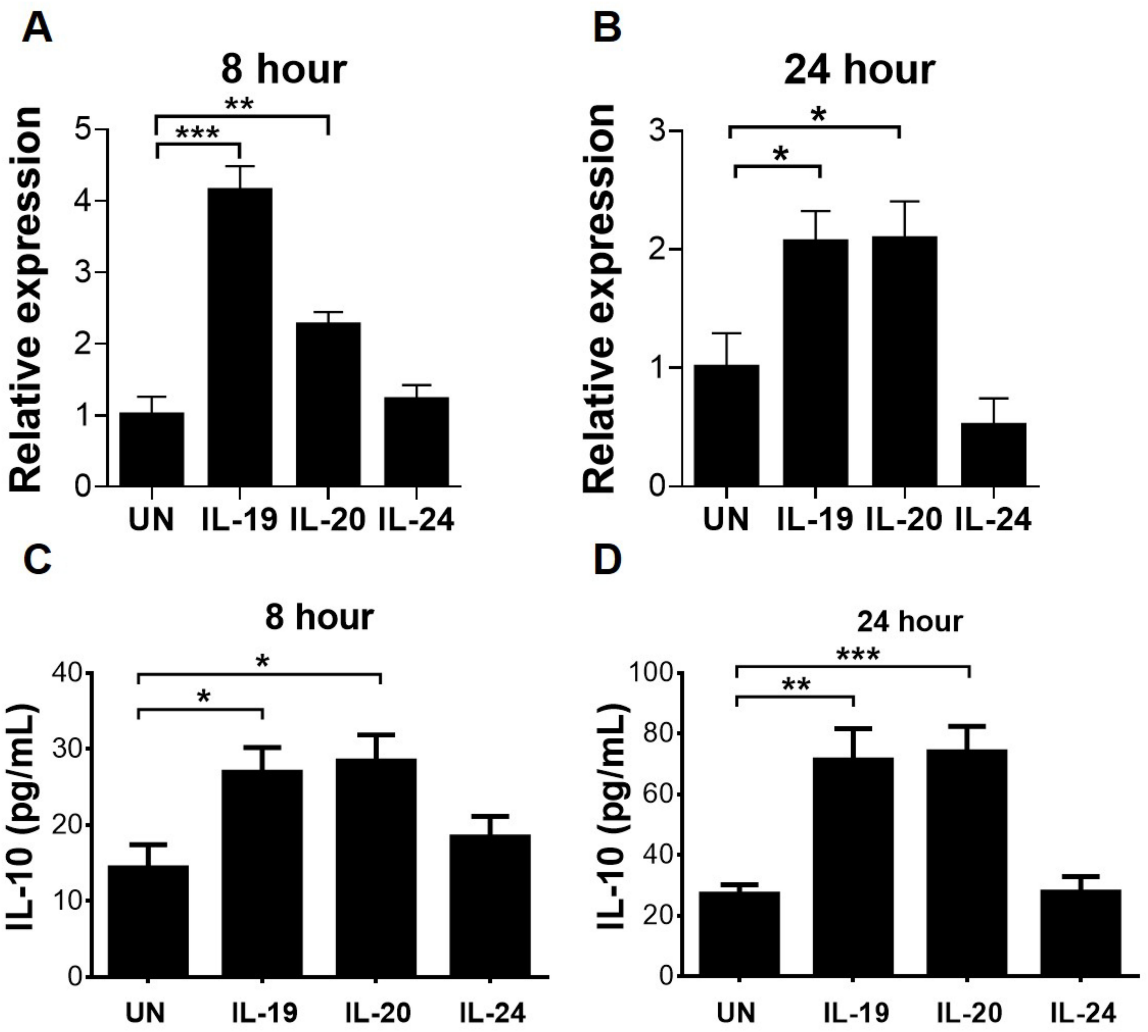
Effects of IL-20RA cytokines on mRNA and protein levels of IL-10 in CD4 T cells. **(A, B)** CD4 T cells harvested from uninfected WT mice were treated without (UN) or with the indicated cytokines for 8 or 24 hours and centrifuged. Total RNA isolated from the cell pellet was subjected to quantitative real-time RT-PCR. The *Il10/β-actin* mRNA levels are shown, and the levels of control samples without cytokine treatment were set as 1. **(C, D)** The culture supernatants were collected to measure IL-10 by ELISA. Data show means + SEM of 6 samples per group. *P < 0.05; **P < 0.01; ***P < 0.001.

Our *in vivo* results showed that the percentage of T cells expressing IL-10 was elevated in infected WT mice when compared to infected *IL-20RA*^-/-^ mice (**Figure 7C**). A mouse *in vitro* study showed that IL-19 treatment increases IL-10 production in CD4 T cells (21). We therefore performed *in vitro* studies to investigate the effect of IL-20RA cytokines on mouse CD4 T cells to express IL-10. Mouse CD4 T cells were harvested from uninfected WT mice with 90% purity and treated with IL-19, IL-20, or IL-24. Our results showed that treatment with IL-19 or IL-20, but not IL-24, for 8 or 24 hours significantly enhanced the mRNA and protein levels of IL-10 in CD4 T cells (**Figure 8**).

**Figure 9:**
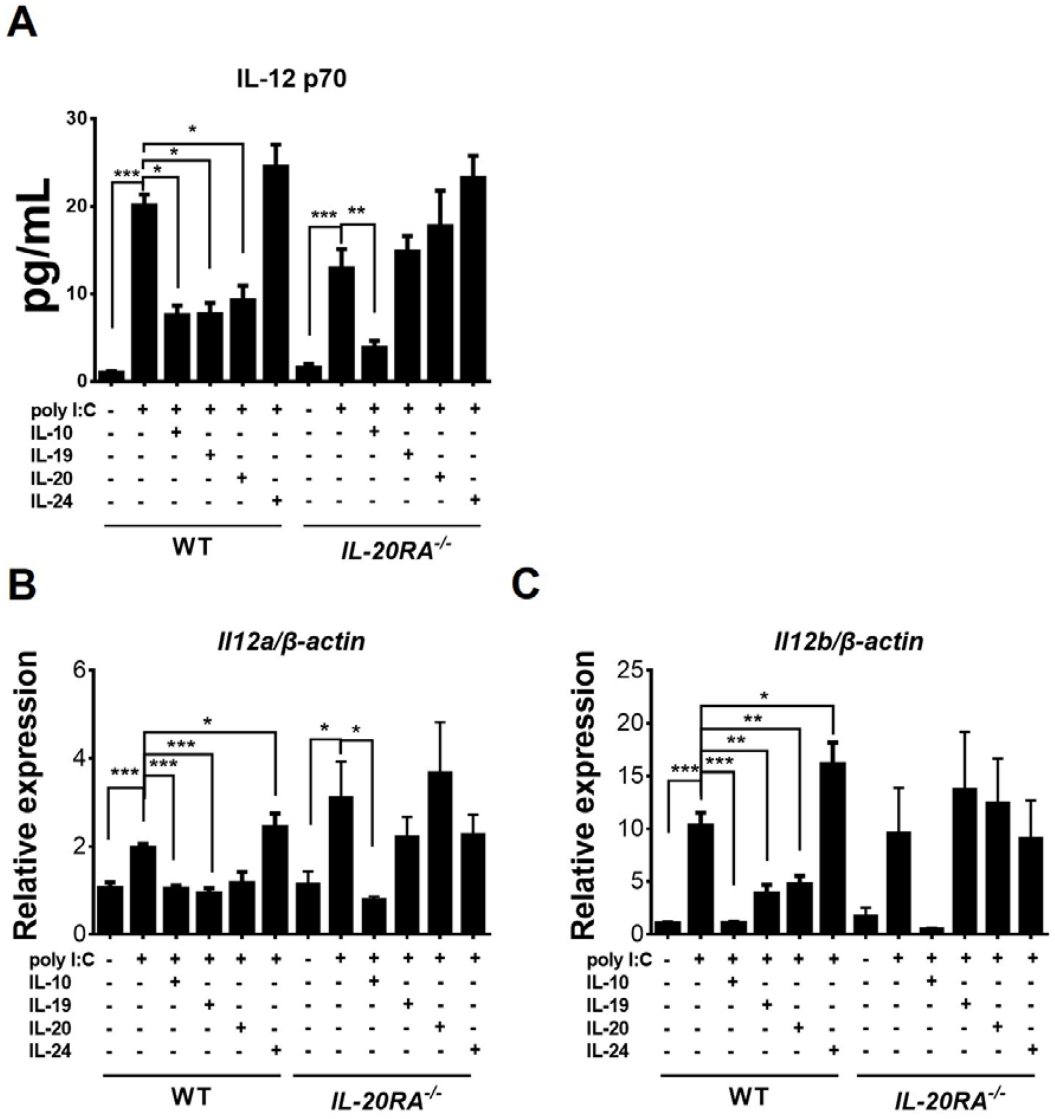
Effects of IL-20RA cytokines on protein and mRNA levels of IL-12 in macrophages. Peritoneal macrophages were harvested from uninfected WT or *IL-20RA*^-/-^ mice, treated without **(-)** or with the indicated cytokine for 3 days, stimulated without **(-)** or with poly I:C for 24 hours, and centrifuged. **(A)** The culture supernatant was collected to measure IL-12 by ELISA. Total RNA isolated from the cell pellet was subjected to quantitative real-time RT-PCR. Levels of *Il12a/β-actin****(*B*)*** and *Il12b/β-actin***(C)** are shown. The levels of control samples without cytokine and poly I:C treatment were set as 1. Data show means + SEM of 6 samples per group. *P < 0.05; **P < 0.01; ***P < 0.001.

Our *in vivo* results showed that abundant macrophages expressed IL-12 in infected *IL-20RA*^-/-^ mice and that the percentage of macrophages expressing IL-12 was reduced in infected WT mice when compared to infected *IL-20RA*^-/-^ mice (**Figure 5C**). We further assessed whether IL-20RA cytokines suppress IL-12 in macrophages by *in vitro* study, as few studies have investigated this issue. IL-12 is a 70-kDa heterodimeric cytokine composed of two subunits, p35 and p40 (40), encoded by the mouse genes *Il12a* and *Il12b*, respectively. We harvested peritoneal macrophages from mice for studies, and more than 90% of the cells were positive for F4/80, a marker specific for mouse macrophages, by immunofluorescence staining (data not shown) as previously described (12). Our ELISA results showed that a very low level of IL-12 p70 protein was detected in the culture supernatant of unstimulated macrophages obtained from WT mice (**Figure 9A**). To boost the IL-12 level in macrophages, we infected macrophages with EV-A71. The virus failed to boost IL-12, probably because it failed to infect and enter macrophages. We then assessed the RNA virus mimic, poly I:C, which is reported to enhance IL-12 in mouse bone marrow–derived macrophages (41). We measured p70 protein by ELISA as well as *Il12a* and *Il12b* mRNA by a quantitative real-time RT-PCR assay. Stimulation with poly I:C for 24 hours enhanced the levels of *Il12a* and *Il12b* mRNA as well as IL-12 p70 protein in macrophages obtained from WT or *IL-20RA*^-/-^ mice (**Figure 9**). IL-10 is reported to suppress IL-12 (17, 18) and was therefore used as a control for the assay. Macrophages were treated with cytokines for 3 days, stimulated with poly I:C for 24 hours, and harvested for assays. IL-10 treatment reduced the levels of *Il12a* and *Il12b* mRNA as well as IL-12 p70 protein in macrophages obtained from WT or *IL-20RA*^-/-^ mice (**Figure 9**). Treatment with IL-19 or IL-20, but not IL-24, decreased the levels of *Il12a* and *Il12b* mRNA as well as IL-12 p70 protein in macrophages obtained from WT mice, but not in those from *IL-20RA*^-/-^ mice (**Figure 9**).

### IL-19 is detected in the plasma of healthy controls, and EV-A71 infection increases plasma IL-19 levels in patients

**Figure 10:**
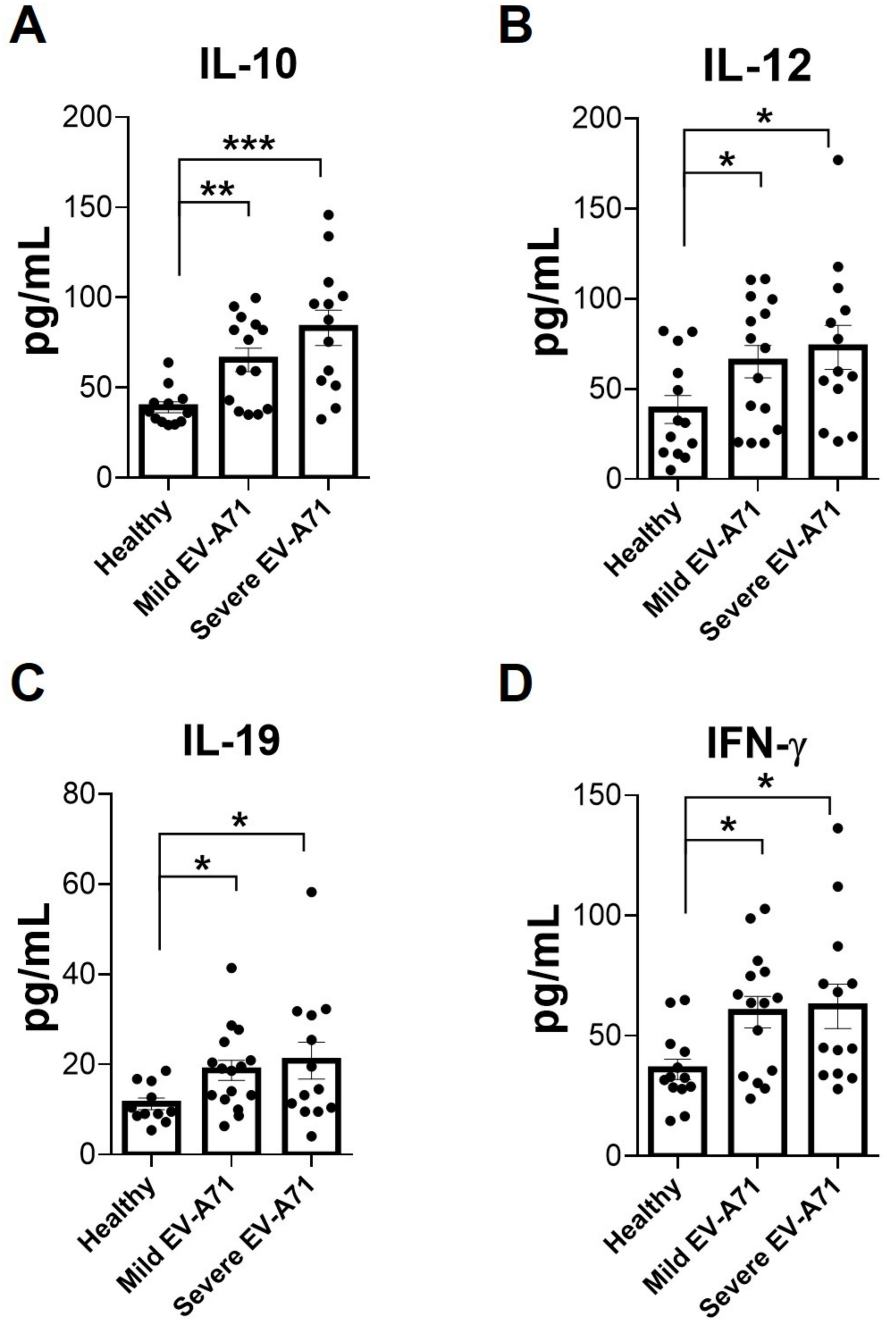
Cytokine levels in the plasma of healthy donors and EV-A71 patients. Plasma samples collected from EV-A71-infected children with mild or severe symptoms and healthy donors were assayed for IL-10, IL-12, IL-19, and IFN-γ **(A-D)** by ELISA. Data show means + SEM of ≥13 samples in each group. *P < 0.05; **P < 0.01; ***P < 0.001.

As both IL-19 and IL-20 enhance IL-10 in mouse CD4 T cells but suppress IL-12 in mouse macrophages *in vitro*, we measured these four cytokines and IFN-γ in the plasma specimens collected from patients who tested positive for EV-A71 and from healthy controls. EV-A71-infected patients were divided into two groups: one with severe symptoms, brainstem encephalitis with or without pulmonary edema, and the other with mild symptoms, such as fever, herpangina, or hand-foot-and-mouth disease. IL-19 was detected in healthy controls (**Figure 10**). The IL-19 levels of infected patients with severe or mild symptoms were higher than those of healthy controls. The IL-19 level of EV-A71-infected patients with severe symptoms was slightly higher than that of patients with mild symptoms. The results of IL-10, IL-12, and IFN-γ were similar to those of IL-19 (**Figure 10**). The IL-20 levels of both infected patients and healthy controls were below detection.

## Discussion

**Figure 11:**
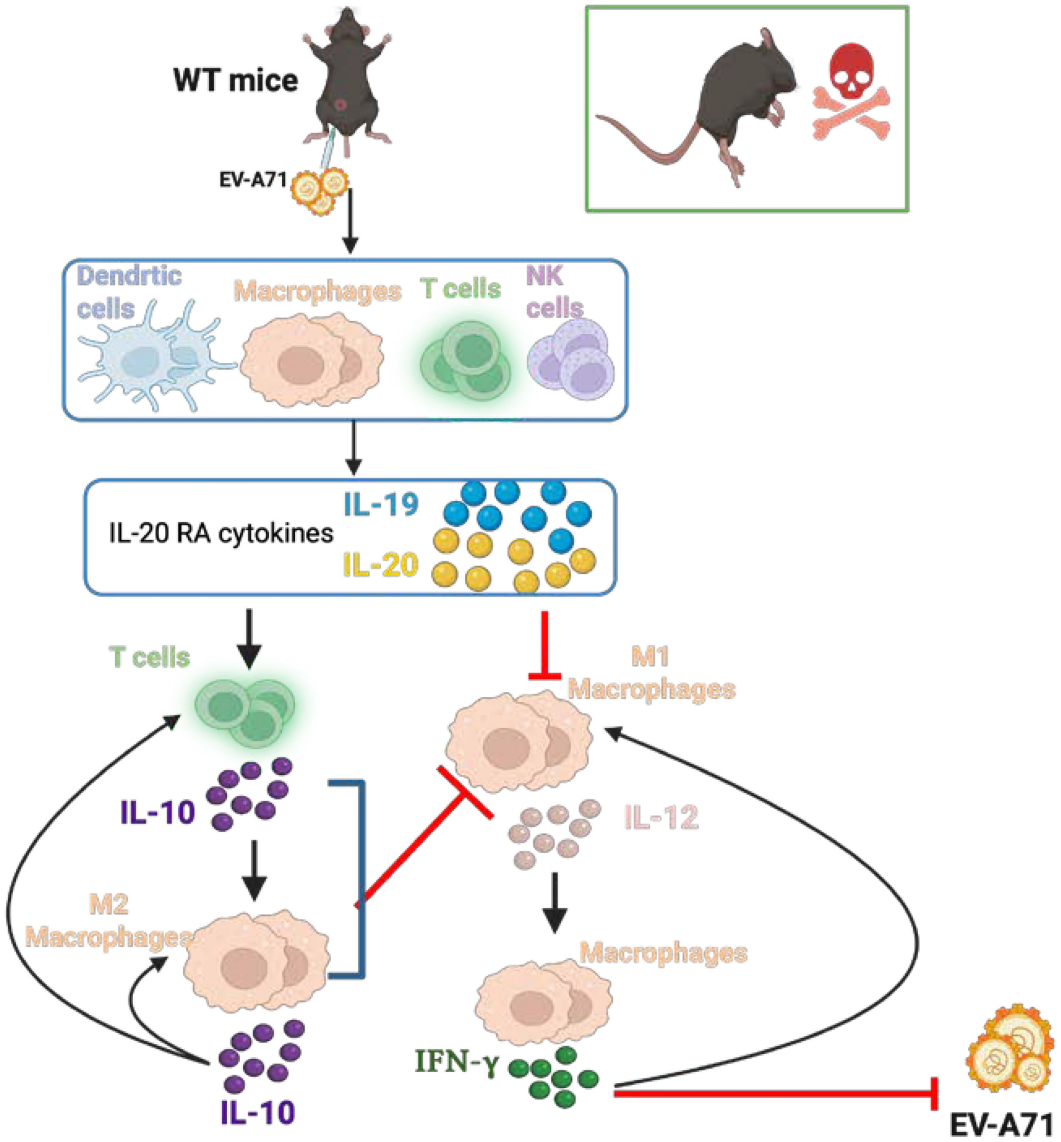
The mechanism of IL-20RA cytokines aggravating EV-A71 infection. EV-A71 infection enhances leukocytes to express IL-20RA cytokines, which increase T cells to produce IL-10 to promote M2 macrophages and suppress M1 macrophage-IL-12-IFN-γ axis to result in increases of viral replication and lethality in WT mice.

Very few reports investigate the interaction of IL-20RA cytokines with viral infections. Our studies show that EV-A71 infection enhances IL-20RA cytokines, especially IL-19, in patients and in a murine model. The summary of mouse results is shown in **Figure 11**. More importantly, IL-20RA cytokines function to exacerbate EV-A71 infection in WT mice. IL-20RA cytokines promote T cells to produce IL-10, which polarizes macrophages toward the M2 phenotype to reduce levels of M1 macrophages as well as IL-12 and IFN-γ expressed by macrophages. Notably, both IL-19 and IL-20 suppress IL-12 in macrophages, which protects mice from EV-A71 infection. As all three mouse IL-20RA cytokines are constitutively expressed and IL-10 is induced after infection, this suggests that both IL-19 and IL-20 could be the upstream effectors to induce IL-10. These results are unreported before and are novel. Our previous and present mouse studies using IFN-γ receptor–deficient mice and anti–IFN-γ antibody, respectively, showed that IFN-γ protects mice from EV-A71 infection (11). The present study finds that macrophages produce IFN-γ to fight EV-A71 infection in mice.

Leukocytes, especially myeloid cells, are the primary sources of IL-19 and IL-20 (22), so we performed flow cytometry by staining leukocyte markers on the cell surface and the cytokines inside the cells to quantify splenocytes expressing IL-19 or IL-20 in infected WT mice on 3 and 5 d.p.i., the time points at which EV-A71 infection significantly and slightly increased serum IL-19 and IL-20 levels, respectively. CD45^+^IL-19^+^ cells (leukocytes expressing IL-19) were detected in both mock-infected and infected mice, with a slightly increased level of CD45^+^IL-19^+^ cells found in infected mice on 3 d.p.i. when compared to mock-infected mice (**Supplementary Figure S5A**). CD45^+^IL-20^+^ cells (leukocytes expressing IL-20) were detected in infected mice on 3 and 5 d.p.i. and in mock-infected mice (**Supplementary Figure S5B**). These results are consistent with the detection of both IL-19 and IL-20 in the serum of mock-infected and infected mice. Macrophages and dendritic cells are reported to be the cellular sources of IL-19 and IL-20, respectively (22). We found that a high percentage of dendritic cells, followed by macrophages, T cells, and NK cells, expressed IL-19 in infected mice on 3 d.p.i. and in mock-infected mice (**Supplementary Figure S5C**). A high percentage of dendritic cells, followed by NK cells, macrophages, and T cells, expressed IL-20 in infected mice on 3 and 5 d.p.i. and in mock-infected mice (**Supplementary Figure S5D**). These results suggest that dendritic cells might be the main source (major producers) of IL-19 and IL-20. Cells other than leukocytes can express IL-19 (22). EV-A71 infection is reported to induce the activation of NF-κB signaling (42), which is shown to upregulate IL-19 expression in the airway epithelia of asthmatic patients (43). Future studies are needed to find the signaling pathway regarding how EV-A71 infection increases IL-19.

Our additional study also tested a 10-fold lower dose of viral inoculum (1 × 10^5^ PFU/mouse) and obtained similar results as those of high viral dose (1 × 10^6^ PFU/mouse), showing that IL-20RA deficiency ameliorates EV-A71 infection in mice (**Supplementary Figure S6**). We used the high viral dose for study, as the differences in IL-10, IL-12, and IFN-γ levels between infected WT and *IL-20RA*^-/-^ mice are readily detected. Clinical EV-A71 isolates fail to induce death in mice and need to be adapted in mice in order to induce death in 2-week-old mice in our model. The adapted EV-A71 strain fails to induce death in mice older than 2 weeks old. We also studied herpes simplex virus 1 (HSV-1), which can induce death in 6-week-old mice by peripheral (corneal) infection and in 2- or 6-week-old mice by systemic (intraperitoneal) infection (44). In mice infected with HSV-1 by corneal inoculation, virus mainly spreads by the neuronal route and is detected only in the eye, trigeminal ganglia, and brain, but not in other tissues or organs (44). Our additional study found that the survival rate of 2-week-old *IL-20RA*^-/-^ mice infected with HSV-1 by intraperitoneal injection was higher than that of infected WT mice (**Supplementary Figure S7**), in a manner similar to that found in EV-A71-infected mice. However, the survival rates of 6-week-old *IL-20RA*^-/-^ and WT mice infected with HSV-1 by corneal or intraperitoneal inoculation were not statistically significant. The EV-A71 and HSV-1 results show that the important role of IL-20RA cytokines is found in neonatal mice with systemic viral infections, and further studies are needed to address this issue in future.

Previous reports of IL-20RA cytokines mostly focused on T cells to show that IL-19 or IL-20 can increase IL-10 and/or decrease IFN-γ to induce T cell polarization toward a Th2 profile (19–21). We previously found that T cell responses, especially the Th2 responses, promote the production of antibodies, which protect mice from EV-A71 infection (45–47), and that the Th2 cytokine IL-6 decreases EV-A71 lethality of mice (30), showing the protective role of Th2 response in EV-A71 infection of mice. In the present study, we focus on macrophages because of the following results and reasons. IL-20RA cytokines reduce the IL-12/IFN-γ axis in macrophages. Macrophages, but not T cells, are known to be the major IL-12 producers. More abundant macrophages express the protective cytokine IFN-γ than T cells in infected *IL-20RA*^-/-^ mice. Additionally, few studies investigate the effect of IL-20RA cytokines on macrophages until recently (24), as IL-20RA is detected on macrophages (48). Macrophages can differentiate into two distinct subpopulations, classical or inflammatory M1 macrophages and alternative or anti-inflammatory M2 macrophages (49). M1 macrophage differentiation can be induced by Th1 cytokines, such as IFN-γ. M1 macrophages produce cytokines such as IL-12. Although M2 macrophages are more diverse and can be classified into four subtypes depending on the stimuli, the hallmark of all subtypes of M2 macrophages is the secretion of anti-inflammatory cytokine, IL-10 (13, 14). Our *in vivo* results showed that IL-20RA deficiency reduces the level of T cells expressing IL-10 and M2 macrophages but increases the levels of macrophages expressing IL-12 and IFN-γ and M1 macrophages in mice during infection. Consistently, our *in vitro* results showed that treatment of IL-19 or IL-20 enhances IL-10 production in T cells but suppresses IL-12 production in macrophages. IL-20RA cytokines induce STAT3 activation (23), which is shown to enhance IL-10 (50–53) but inhibit IL-12 (54). These findings may explain how IL-20RA cytokines increase IL-10 in T cells but suppress IL-12 in macrophages. Our previous study used the anti-IL-20RA monoclonal antibody (51D) to ameliorate liver damage (fibrosis) in mice (55). Here we show that IL-20RA cytokines aggravate EV-A71 infection in mice. Our additional study tested 51D to reduce EV-A71 infection in mice but failed. As IL-20RA is detected on leukocytes such as macrophages (48), 51D treatment may deplete the protective leukocytes and result in the failure to reduce EV-A71 infection. Future studies can design small molecules targeting IL-20RA and receptor signaling to test the potential of blocking IL-20RA to reduce viral infections. Our study is novel in showing that EV-A71 infection enhances IL-20RA cytokine levels in humans and mice. More importantly, IL-20RA cytokines aggravate viral infection with the elevated axis of T cell–IL-10–M2 macrophage to suppress the protective axis of M1 macrophage–IL-12–macrophage–IFN-γ in mice.

## Data Availability

The original contributions presented in the study are included in the article/**Supplementary Material**. Further inquiries can be directed to the corresponding authors.
