# Supplementary material for: Deficiency of IL-20 receptor subunit A decreases enterovirus A71 lethality in mice by increasing M1 macrophage polarization and cytokine production
Source: Front Immunol. 2026 Jan 2;16:1700154. doi: 10.3389/fimmu.2025.1700154 (PMC12808429; doi:10.3389/fimmu.2025.1700154)
Supplement: Supplementary file 1 [file DataSheet1.pdf]

Supplementary material

SUPPLEMENTARY FIGURES S1-S7

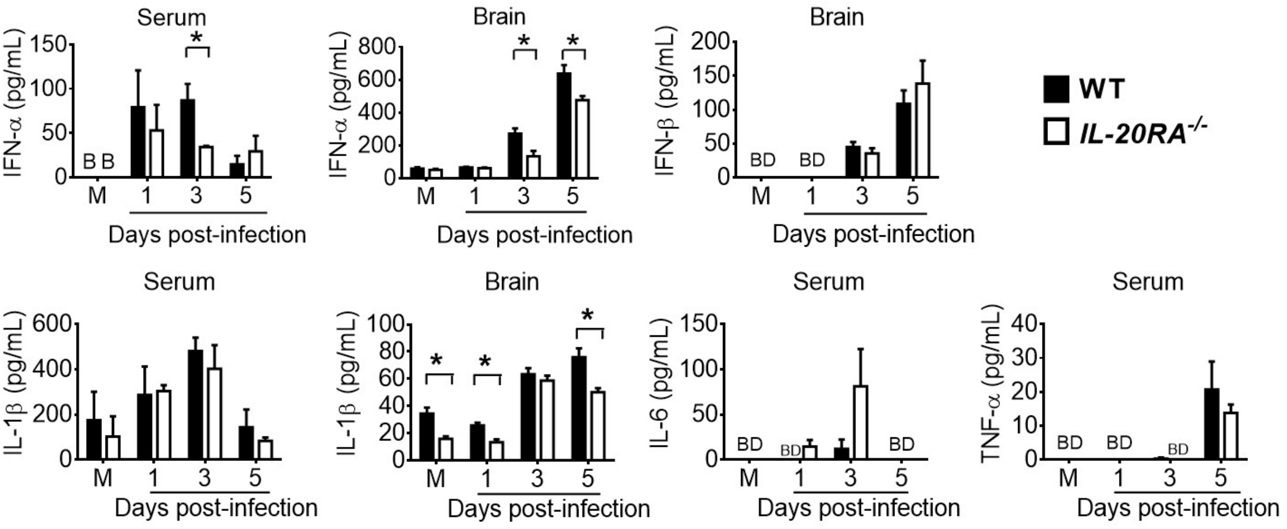

SUPPLEMENTARY FIGURE S1

The effects of IL-20RA deficiency on cytokine levels of mice. The sera and brains of WT mice and *IL-20RA*$^{-/-}$ mice infected with EV-A71 for indicated days or mock-infected (M) were harvested to measure IFN-α, IFN-β, IL-1β, IL-6, and TNF-α by commercially available ELISA kits (R&D Systems) with the detection limits of 3.8, 1.9, 4.8, 1.8, and 7.2 pg/mL. Data show means + SEM of ≥3 samples per data point. *, $P < 0.05$. B or B.D. stands for below the detection limit. The levels of serum IFN-β and brain IL-6 and TNF-α of mock-infected and infected WT mice and *IL-20RA*$^{-/-}$ mice on 1, 3, and 5 days post-infection were below detection.

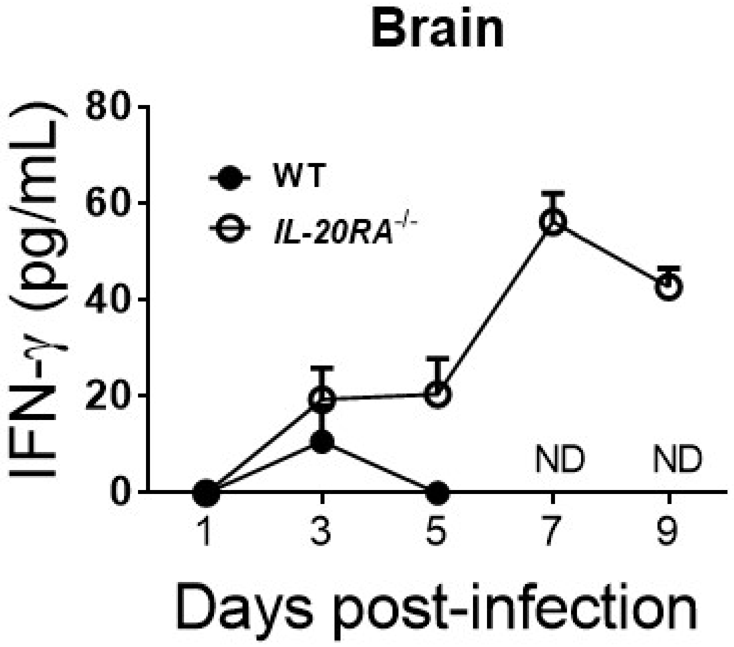

SUPPLEMENTARY FIGURE S2

The effect of IL-20RA deficiency on brain IFN-γ levels of infected mice. The brains of WT mice and *IL-20RA*$^{-/-}$ mice infected with EV-A71 for indicated days were harvested to measure IFN-γ by ELISA. Data show means + SEM of ≥4 samples per data point. ND stands for "not done", because samples were unavailable due to mouse death.

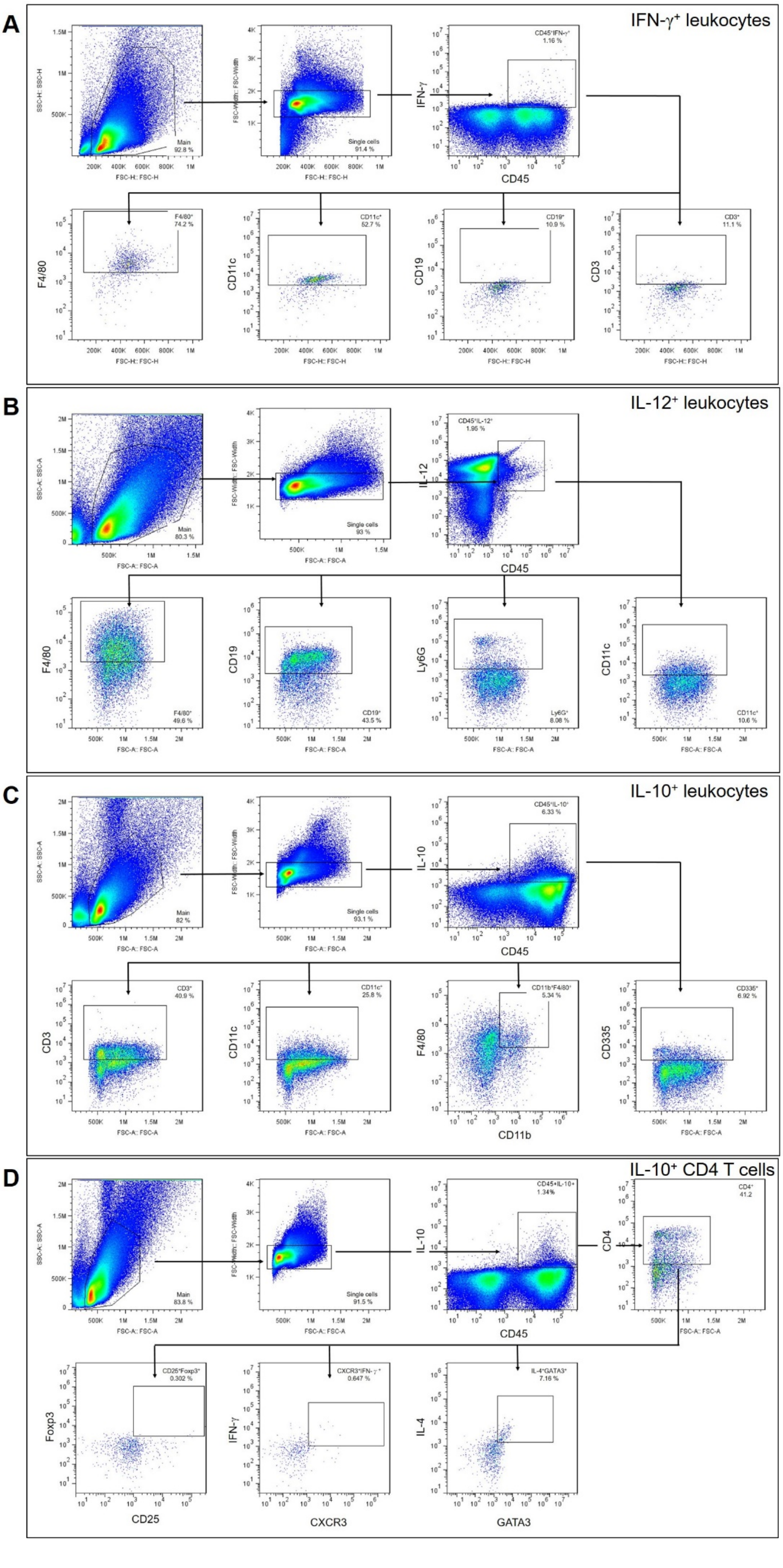
A
IFN-γ+ leukocytes
Main 92.8 %
Single cells 91.4 %
CD45+IFN-γ+ 1.16 %
IFN-γ
CD45
F4/80+ 74.2 %
CD11c+ 52.7 %
CD19+ 10.9 %
CD3+ 11.1 %
F4/80
CD11c
CD19
CD3
B
IL-12+ leukocytes
Main 80.3 %
Single cells 93 %
CD45+IL-12+ 1.95 %
IL-12
CD45
F4/80
CD19
Ly6G
CD11c
F4/80+ 49.6 %
CD19+ 43.5 %
Ly6G+ 8.08 %
CD11c+ 10.6 %
C
IL-10+ leukocytes
Main 82 %
Single cells 93.1 %
CD45+IL-10+ 6.33 %
IL-10
CD45
CD3+ 40.9 %
CD11c+ 25.8 %
CD11b+F4/80+ 5.34 %
CD335+ 6.92 %
CD3
CD11c
F4/80
CD11b
CD335
D
IL-10+ CD4 T cells
Main 83.8 %
Single cells 91.5 %
CD45+IL-10+ 1.34 %
IL-10
CD45
CD4+ 41.2
CD4
CD25+Foxp3+ 0.302 %
CXCR3+IFN-γ+ 0.647 %
IL-4+GATA3+ 7.16 %
Foxp3
IFN-γ
IL-4
CD25
CXCR3
GATA3

SUPPLEMENTARY FIGURE S3

Gating strategy for flow cytometry. (**A)** Gating strategy for F4/80$^+$ (macrophage), CD11c$^+$ (dendritic cell), CD19$^+$ (B cell), or CD3$^+$ (T cell) cells of CD45$^+$IFN-γ$^+$ leukocytes from mouse splenocytes. (**B)** Gating strategy for F4/80$^+$ (macrophage), CD19$^+$ (B cell), Ly6G$^+$ (neutrophil), or CD11c$^+$ (dendritic cell) cells of CD45$^+$ IL-12$^+$ leukocytes from mouse splenocytes. (**C)** Gating strategy for CD3$^+$ (T cell), CD11c$^+$ (dendritic cell), F4/80$^+$ (macrophage), or CD335$^+$ (NK cell) cells of CD45$^+$IL-10$^+$ leukocytes from mouse splenocytes. (**D)** Gating strategy for Foxp3$^+$ CD25$^+$ (Treg), IFN-γ$^+$CXCR3$^+$ (Th1), or IL-4$^+$GATA3$^+$ (Th2) cells of CD45$^+$IL-10$^+$CD4$^+$ T cells from mouse splenocytes. Antibodies against CD4 (clone GK1.5, BD Bioscience), CD25 (clone 3C7, BD Bioscience), Foxp3 (clone MF-14, Biolegend), CXCR3 (clone CXCR3-173, Biolegend), or GATA3 (clone 16E10A23, Biolegend) were used.

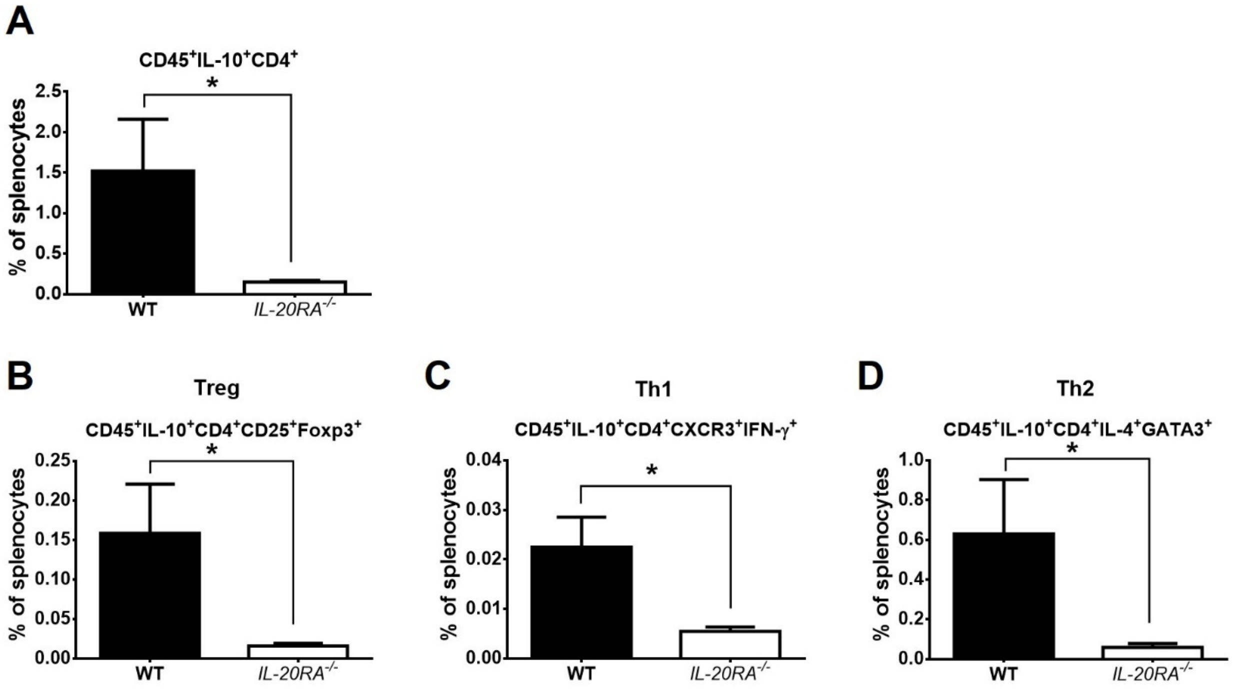

SUPPLEMENTARY FIGURE S4

IL-20RA deficiency decreases IL-10-expressing CD4 T cells of EV-A71-infected mice. Splenocytes of WT and *IL-20RA*$^{-/-}$ mice infected with EV-A71 were harvested on day 1 post-infection and stained for IL-10 inside cells and the indicated leukocyte markers on membrane (**A**) and for Treg (**B**), Th1 (**C**), or Th2 (**D**) cells. Data show means + SEM of 6 samples per group. *, $P < 0.05$.

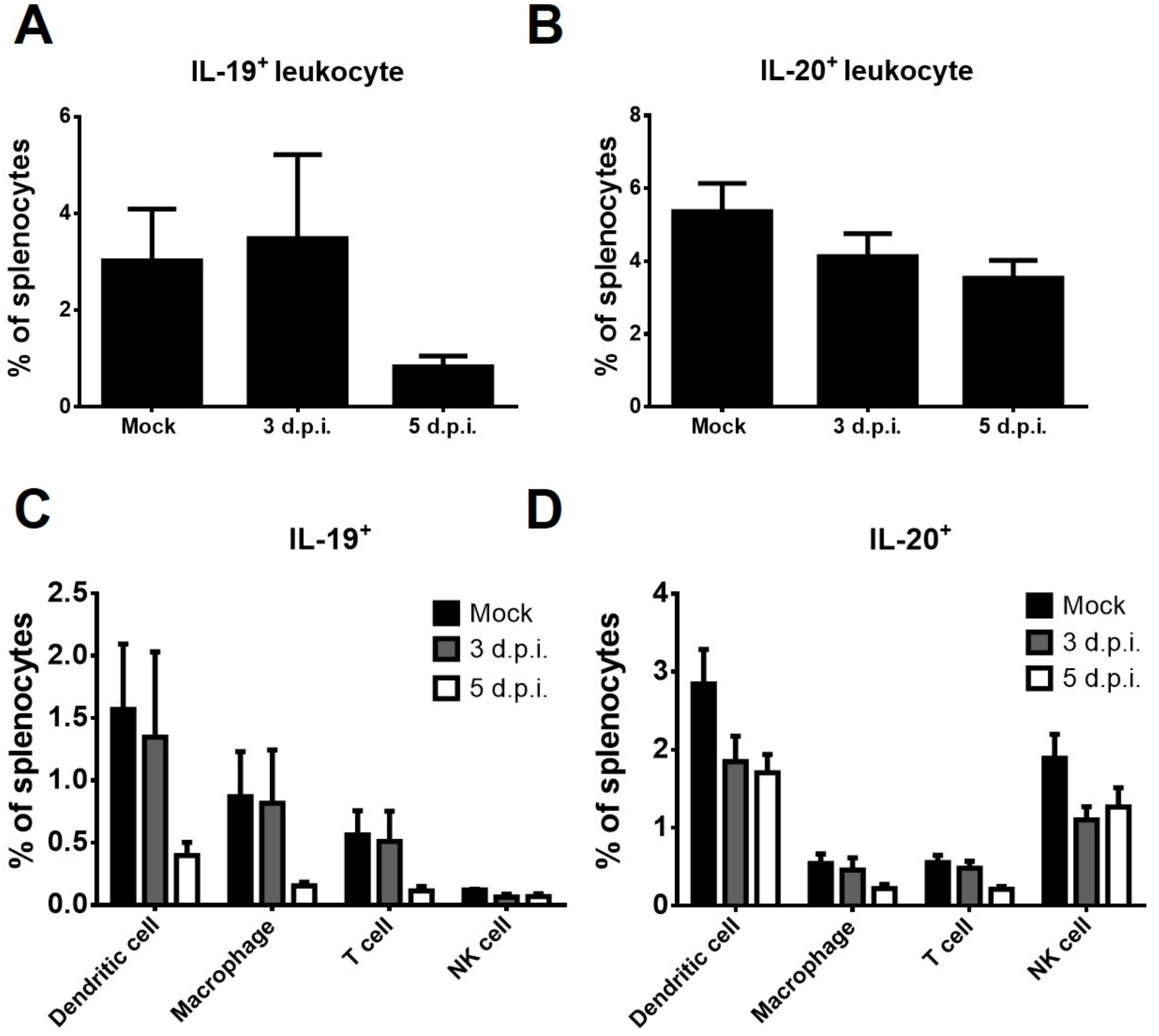

SUPPLEMENTARY FIGURE S5

Leukocytes expressing IL-19 or IL-20 in mice. The spleens of mock-infected or infected mice were collected, processed into splenocytes, and stained with antibodies for IL-19 (clone 350105, R&D Systems) or IL-20 (clone 7E, provided by Dr. Ming-Shi Chang) and leukocyte markers, CD45 plus dendritic cells, macrophages, T cells, or NK cells. The percentages of IL-19$^+$ leukocytes (**A**), IL-20$^+$ leukocytes (**B**), IL-19$^+$ leukocyte subsets (**C**), and IL-20$^+$ leukocyte subsets (**D**) are shown. Data are means + SEM of 3 samples per group.

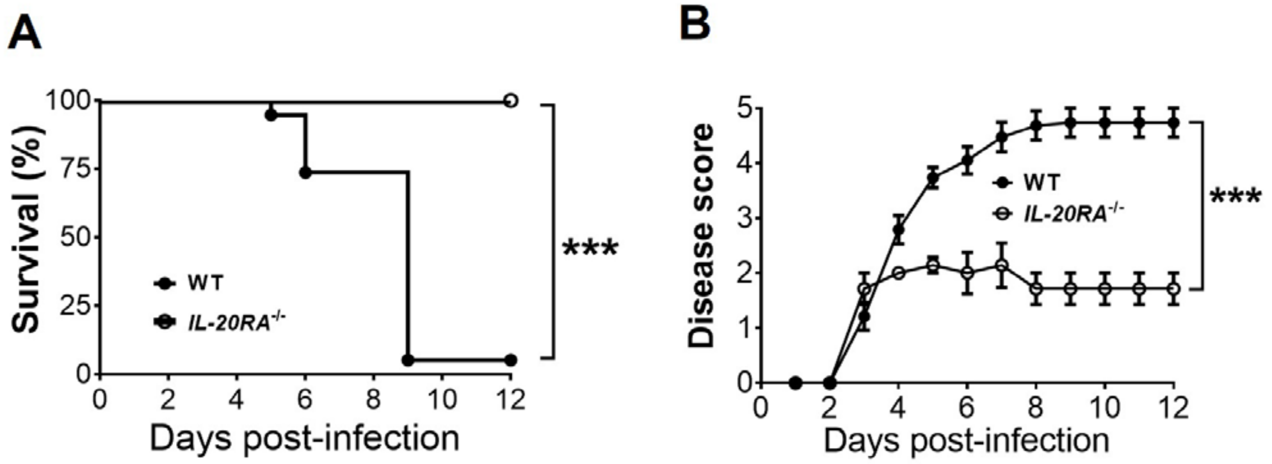

SUPPLEMENTARY FIGURE S6

IL-20RA deficiency ameliorates EV-A71 infection in mice. The survival rates (**A**) and disease scores (**B**) of WT and *IL-20RA*$^{-/-}$ mice infected with EV-A71 at the dose of 1×10^5 PFU/mouse are shown ($n \geq 7$ per group). ***, $P < 0.001$.

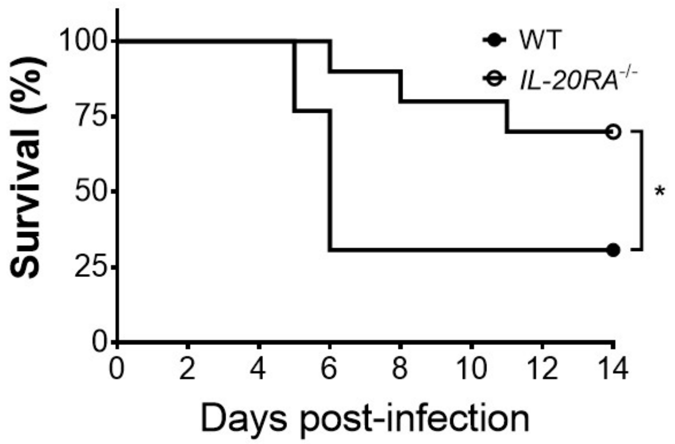

SUPPLEMENTARY FIGURE S7

IL-20RA deficiency decreases HSV-1 lethality of mice. The survival rates of 2-week-old WT and *IL-20RA*$^{-/-}$ mice infected with HSV-1 strain RE by intraperitoneal injection are shown ($n \geq 6$ in each group). *, $P < 0.05$.
